# Genomic diversity and clade clustering of *Burkholderia pseudomallei* and *B. thailandensis* prophages with soil-derived phages

**DOI:** 10.1016/j.isci.2026.114658

**Published:** 2026-01-09

**Authors:** Patoo Withatanung, Veerachat Muangsombut, Sujintana Janesomboon, Vanaporn Wuthiekanun, Premjit Amornchai, Sorujsiri Chareonsudjai, Dave J. Baker, Martha R.J. Clokie, Edouard E. Galyov, Ozan Gundogdu, Sunee Korbsrisate

**Affiliations:** 1 Department of Immunology, Faculty of Medicine Siriraj Hospital, Mahidol University, Bangkok, Thailand; 2 Mahidol-Oxford Tropical Medicine Research Unit, Faculty of Tropical Medicine, Mahidol University, Bangkok, Thailand; 3 Department of Microbiology, Faculty of Medicine, Khon Kaen University, Khon Kaen, Thailand; 4 Science Operations, Quadram Institute Bioscience, Norwich Research Park, Norwich, UK; 5 Becky Mayer Centre for Bacteriophage Research, Department of Genetics, Genomics and Cancer Sciences, University of Leicester, Leicester, UK; 6 Department of Infection Biology, Faculty of Infectious and Tropical Diseases, London School of Hygiene and Tropical Medicine, London, UK

**Keywords:** Agricultural soil science, Microbiology, Natural sciences, Soil ecotoxicology

## Abstract

Most studies on bacteriophages (phages) of the Gram-negative bacterium *Burkholderia pseudomallei* rely on *in silico* predictions and thus underestimate the true diversity of phages. Analysis of the whole genome sequences of culturable prophages induced from *B. pseudomallei* and *B. thailandensis*, along with their free *Burkholderia* phages isolated from soils in Thailand, identified six novel groups of *Burkholderia* phages, surpassing *in silico* expectations. The analysis also indicated that soil-dwelling phages may have originated from lysogenic *B. pseudomallei* strains. Free phages isolated from soil showed high nucleotide similarity to prophage sequences in *B. pseudomallei*, including phages previously cultured from melioidosis patients’ hemocultures, indicating that similar phage types occur in both environmental and clinical sources. Phylogenomic analysis also revealed close genomic relatedness between prophages from *B. thailandensis* and *B. pseudomallei*, although the biological significance remains unknown. Together, these findings refine our understanding of the genomic diversity and ecological patterns of *Burkholderia* phages.

## Highlights

• Genome analysis of 82 culturable *Burkholderia* phages revealed novel phage clusters
• Soil-dwelling phages may have originated from lysogenic *B. pseudomallei* strains
• Blood-isolated phages linked to prophage sequences in the *B. pseudomallei* genome
• Close genetic similarity among *B. pseudomallei*, *B. thailandensis*, and BTCV prophages

## Graphical abstract

**Figure:**
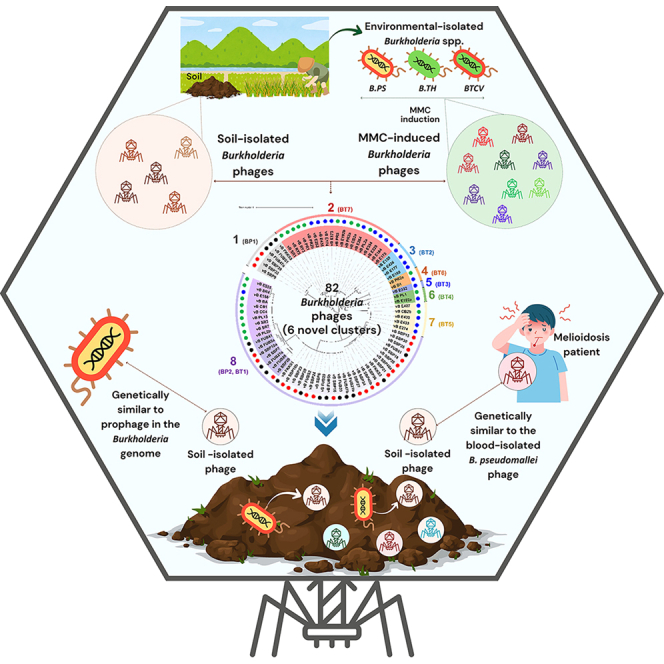

## Introduction

The genus *Burkholderia* comprises bacteria with diverse ecological and clinical roles, ranging from environmental symbionts to highly virulent pathogens. *Burkholderia pseudomallei*, a Gram-negative bacterium, is classified as a virulent pathogen that causes a life-threatening tropical infectious disease called melioidosis. The disease is endemic in Northern Australia and Southeast Asia, with an estimated 165,000 infections annually,^1^ and mortality rates reaching 40%.^2^ *B. pseudomallei* is a soil saprophyte commonly found in soil and water in endemic areas.^3^ However, the environmental conditions that govern its persistence in soil and water remain unclear.

Humans and animals become infected through contact with contaminated soil or water (especially through skin abrasions/wounds) or via air.^4^ Unlike *B. pseudomallei*, *Burkholderia thailandensis* is less pathogenic for humans, and most *B. thailandensis* isolates do not produce a capsule, a recognized virulence determinant of *B. pseudomallei*.^5^ Interestingly, a capsulated *B. thailandensis* strain that produces *B. pseudomallei*-like capsules (BTCV) was recently discovered, highlighting their close genetic similarity.^6^ In Thailand, where melioidosis is endemic, *B. pseudomallei* and *B. thailandensis* are uncommon in the same fields. *B. pseudomallei* is predominantly isolated in the Northeast, whereas *B. thailandensis* is more commonly found in the East and Central regions, although it is also present in the Northeast too. BTCV is uncommon, but is closely associated with *B. thailandensis*.^6^

Lysogeny is a state in which a bacteriophage (phage) persists within a bacterial host, either through integration into the host chromosome or by maintaining itself as an extrachromosomal element.^7^ Such integrated phages are known as prophages or temperate phages, and they often encode genes that provide advantages to their host, such as improved fitness, enhanced virulence, and adaptability to challenging environments.^8^^,^^9^ Lysogens (bacteria that carry prophage) can remain in the lysogenic cycle for many generations but can switch to the lytic cycle at any time, leading to bacterial lysis and the release of hundreds of progenies. The decision between lytic and lysogenic cycles is influenced by multiple factors, including host cell stress, metabolic state, and phage regulatory networks.^10^ Most *B. pseudomallei* and *B. thailandensis* strains are lysogenic, and mulitple prophages exist in their genomes.^11^ Interestingly, temperate phages from *B. pseudomallei* can also infect and integrate into *B. thailandensis* where they induce phenotypic changes in infected strains, such as increased serum resistance.^12^ The significance of prophage abundance, diversity, and their role in *Burkholderia* spp. pathogenesis and survival in the environment warrants further investigation.

Indeed, our previous work showed that free phages could be isolated from the environment. The observed *Burkholderia* podovirus AMP1 was isolated from soil in a melioidosis-endemic region in Thailand.^13^ Subsequent analyses suggested that its temperature-dependent characteristics may influence both *B. pseudomallei* virulence and detectability in environmental and clinical samples.^14^ Moreover, Yordpratum et al.^15^ identified six *Burkholderia* myoviruses from soil samples, indicating the presence of *Burkholderia* phage population in soil environments. Furthermore, our past research revealed that *B. pseudomallei* and its free phages could be co-isolated from soil samples.^16^

Not only from environmental sources, phages can be identified in clinical samples. Recent research study demonstrated that *B. pseudomallei* phages could be cultured from hemoculture-confirmed melioidosis patients. Data suggest that these phages are induced from bacteria within circulating blood.^17^ These blood-isolated phages carry putative bacterial virulence genes such as virulence-associated protein E (*vapE*), suggesting they may play a role in modulating *B. pseudomallei* virulence.^17^ The specific factors that trigger prophage induction from the *B. pseudomallei* genome remain unclear. Additionally, the role of prophages in *Burkholderia* adaptation to environmental conditions and human pathogenesis is yet to be fully understood. At present, most studies on *Burkholderia* prophages rely on *in silico* analysis. Since not all *Burkholderia* isolates undergo whole-genome sequencing, many natural strains may harbor uncharacterized prophages. Environmental prophage diversity likely exceeds current *in silico* predictions, as shown by spontaneous prophage induction studies in environmental bacteria.^18^ Isolation and whole-genome sequencing of prophages induced from *Burkholderia* and their free phages collected from soil in the endemic area of melioidosis are essential for elucidating their diversity, origin, and the relationships between phages in environmental and clinical settings, as well as identifying genetic determinants that may contribute to bacterial adaptation, environmental persistence, and pathogenic potential.

To address this, soil samples were collected from the northeast of Thailand where melioidosis is endemic for the isolation of both *B. pseudomallei* and their phages. Additionally, *B. thailandensis*, and BTCV were collected from soils in the central and northeast of Thailand. A total of 100 *Burkholderia* bacteria were treated with mitomycin C (MMC) to induce prophages; this yielded 66 culturable prophages. These prophages together with 16 free culturable *Burkholderia* phages isolated from soil were analyzed by whole-genome sequencing. Comparative genomic analyses mapped the prophages and free phages into various clades, revealing substantial *Burkholderia* phage diversity and distinct evolutionary pathways. Remarkably, a novel *B. pseudomallei* phage group was identified, indicating that environmental prophage diversity exceeds current *in silico* predictions. This study expands current understanding of *Burkholderia* phage diversity and highlights genomic relationships between phages from environmental sources and those previously detected in clinical isolates.

## Results

### *B. pseudomallei* and their phages are prevalent in soil of endemic area

**Figure 1:**
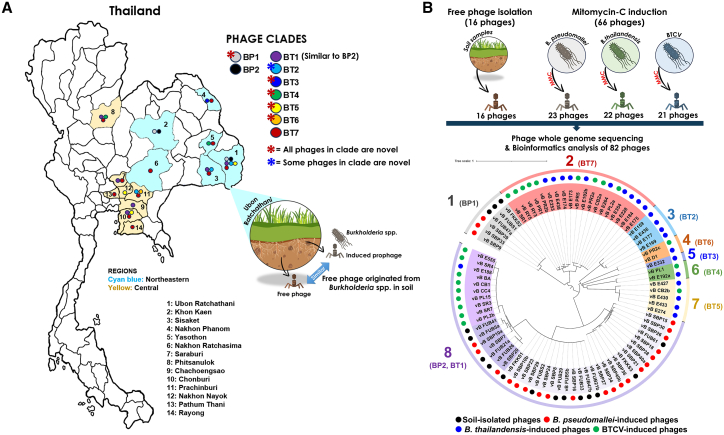
Genome-wide comparative analysis and clade classification of 82 *Burkholderia* phages (A) Map of Thailand showing the geographic distribution of *Burkholderia* phage clades across different provinces. *Burkholderia* phages are classified into clades BP1-BP2 and BT1-BT7, each represented by distinct colored circles. Cyan blue shading denotes provinces in the Northeastern region, and yellow shading denotes provinces in the Central region. Provinces are numbered (1–14) and listed below the map. *B. thailandensis*-associated phages show greater phage diversity than *B. pseudomallei*-associated phages. Red asterisks indicate that all phages in the clade are novel, while blue asterisks indicate that some phages in the clade are novel. (B) Whole-genome comparative analysis of 82 *Burkholderia* phages, comprising 16 free phages isolated from soil and 66 MMC-induced phages, was used to construct a phylogenetic tree (tree scale = 1 nucleotide substitution per site). Colored dots adjacent to each phage name indicate the isolation source: soil-isolated (black), *B. pseudomallei*-induced (red), *B. thailandensis*-induced (blue), and BTCV-induced (green). The analysis resolved the phages into eight major clusters (numbered 1–8). Clusters 2–7 were composed predominantly of *B. thailandensis*-associated phages (clades BT2-BT7), whereas cluster 1 contained only *B. pseudomallei*-associated phages (clade BP1). Cluster 8 was the largest and most diverse, encompassing both *B. pseudomallei*-associated (clade BP2) and *B. thailandensis*-associated phages (clade BT1).

A total of 100 soil samples were collected from two provinces (Ubon Ratchathani and Khon Kaen provinces), which are ∼287 km apart, in the northeast of Thailand where melioidosis is endemic (Figure 1A). Forty of fifty soil samples collected from Ubon Ratchathani were culture-positive for *B. pseudomallei* (80% isolation rate), whereas none of the 50 soil samples from Khon Kaen province tested positive for *B. pseudomallei* by culture.

In addition, the same 100 soil samples were tested for the presence of *Burkholderia* phages that could form plaques on *B. pseudomallei* isolate UB4. Of the fifty soil samples collected from Ubon Ratchathani province, 10 samples yielded 13 *Burkholderia* phages (20% isolation rate), with three samples yielding two phages each. Among these, 8 soil samples harbored both *B. pseudomallei* and free phages, whereas 2 soil samples contained only free phages. Whereas, 3/50 soil samples from Khon Kaen tested positive for *Burkholderia* phages (6% isolation rate), yielding a total of 16 soil-isolated free phages for further characterization.

### Prophages could be induced from soil-isolated *B. pseudomallei*

The 40 *B. pseudomallei* collected from Ubon Ratchathani were induced using MMC to examine them for phage content. We found that phages could be induced from 21 out of 40 (52.5%) isolates. Among these 21 phage-positive samples, two isolates produced two distinct plaque morphologies, suggesting that these samples contained two different culturable phages. Therefore, a total of 23 MMC-induced *B. pseudomallei* phages were included for further study.

### BTCV showed a higher proportion of prophage induction than *B. pseudomallei* and *B. thailandensis* under our experimental conditions

To compare the MMC-induced prophages from *B. pseudomallei* and related species, 40 *B. thailandensis* and 20 BTCV isolates were also induced using MMC. Prophages could be induced from 21 out of 40 (52.5%) *B. thailandensis* isolates, with one isolate producing two distinct plaque morphologies. Interestingly, 17 out of 20 (85.0%) BTCV isolates were prophage-inducible, with one isolate producing three distinct plaque morphologies and two isolates producing two distinct plaque morphologies. As a result, a total of 43 prophages were obtained, composed of 22 from *B. thailandensis* and 21 from BTCV.

### Induced *Burkholderia* prophages and soil-isolated free phages could be classified into eight distinct groups

To comprehensively assess the diversity and genetic relationships of MMC-induced prophages from *B. pseudomallei* (23 phages), *B. thailandensis* (22 phages), BTCV (21 phages) and 16 soil-isolated culturable phages, whole-genome sequencing and analysis were undertaken. As shown in Tables 1 and S1, the genomes of all 82 *Burkholderia* phages ranged from 33.9 to 58.2 kb and encoded 42 to 86 ORFs. PhageTerm analysis did not detect fixed genome termini in any of the 82 isolates and consistently indicated circularly permuted genomes with terminal redundancy (Table S2). Because these patterns prevent the identification of a unique physical start position, the genomes were subsequently arranged in a standardized orientation based on conserved gene module organization, allowing the consistent visualization and comparison across clades.

**Table 1:** Summary of 82 *Burkholderia* phages analyzed in this study

Clades	Phages^a^	Numbers of phages	Types of phages	Genome sizes	ORFs	% GC	Nucleotide blast against phages	% CV	% ID
BP1	vB_SBP9^S1^, vB_SBP39^S1^, vB_SBP33^S1^	3	MMC-Bps	46654–48001	69–71	57.9–58.1	phiE125	3	85
	vB_FUBS1^S1^, vB_FKKS2^S2^, vB_FUB47a^S1^	3	Free phage						
BP2	vB_SBP20^S1^, vB_SBP7^S1^, vB_SBP10a^S1^	3	MMC-Bps	33895–37648	45–49	64.6–65.3	phiX216	65–94	97.26–99.35
	vB_FUB26^S1^, vB_FUB21a^S1^, vB_FUB5a^S1^, vB_FUB41^S1^	4	Free phage						
	vB_SBP30^S1^, vB_SBP26^S1^, vB_SBP38^S1^, vB_SBP40a^S1^, vB_SBP18^S1^, vB_SBP15^S1^, vB_SBP21^S1^, vB_SBP36^S1^, vB_SBP40b^S1^, vB_SBP34^S1^, vB_SBP27^S1^	11	MMC-Bps	35144–37474	42–50	64.6–65.4	phiE12-2	61–100	95.49–100
	vB_FUB61^S1^, vB_FKKS3^S2^	2	Free phage						
	vB_SBP10b^S1^, vB_SBP23^S1^, vB_SBP24^S1^, vB_SBP8^S1^, vB_SBP29^S1^, vB_SBP16^S1^	6	MMC-Bps	34409–36696	47–49	64.5–64.6	phiE12-2	99–100	99.9–99.96
	vB_FUB21b^S1^, vB_FUB47b^S1^, vB_FUB29^S1^, vB_FUBS2^S1^, vB_FUB5b^S1^, vB_FUB33^S1^, vB_FKKS1^S1^	7	Free phage						
BT1	vB_E188^S3^	1	MMC-Bth	36779–39199	49–50	64.6–65.1	phiX216	79–91	93.49–98.3
	vB_SR4^S4^, vB_E555^S5^, vB_PL2b^S6^, vB_SR7^S4^, vB_SR3^S4^, vB_PL15^S6^, vB_CC4^S7^, vB_CB1^S8^, vB_BA^S1^	9	MMC-BTCV						
BT2	vB_E159^S1^, vB_E436^S9^, vB_E177^S3^, vB_E169^S1^	4	MMC-Bth	44120–45068	67–68	63.7–64.1	phiE131	100	100
							phiPE067	73–75	96.54–98.63
BT3	vB_E332^S10^	1	MMC-Bth	42358	52	65.3	phiBcepC6B	75	86.22
BT4	vB_PL1^S6^	1	MMC-Bth	56536–57352	81	63.9–64.1	phiMagia	40–42	90.1–90.18
	vB_E192a^S11^	1	MMC-BTCV						
BT5	vB_CB2b^S8^	1	MMC-Bth	36108–45829	49–59	62.7–64.1	phiE255	80–91	95.54–100
	vB_E427^S12^, vB_E430^S12^, vB_E433^S12^, vB_E274^S1^	4	MMC-BTCV						
BT6	vB_D1^S13^	1	MMC-Bth	36279–38191	49–50	62.5	phiKS10	70–74	83.26–83.3
	vB_PR2c^S9^	1	MMC-BTCV						
BT7A	vB_E192b^S11^, vB_E264^S14^, vB_E175^S1^, vB_E184^S3^, vB_E228^S1^, vB_E354^S10^	6	MMC-Bth	43711–44671	60–63	61–61.5	phiE264.1	86–100	95.7–100
	vB_PR5^S9^, vB_PR2a^S9^, vB_CB2a^S8^, vB_PL2a^S6^	4	MMC-BTCV						
BT7B	vB_E253^S14^, vB_E438^S15^, vB_E174^S1^, vB_E173^S1^	4	MMC-Bth	52117–58216	70–86	60.8–61.3	phiE125	64–97	95.1–99.97
	vB_SR1^S4^, vB_RY5^S16^, vB_RY3^S16^, vB_PR1^S9^, vB_PR2b^S9^	5	MMC-BTCV						

a Sources of bacteria or free-phage (provinces): ^S1^Ubon Ratchathani; ^S2^Khon Kaen; ^S3^Sisaket; ^S4^Saraburi; ^S5^Cambodia; ^S6^Phitsanulok; ^S7^Chachoengsao; ^S8^Chonburi; ^S9^Prachinburi; ^S10^Nakhon Phanom; ^S11^Yasothon; ^S12^Nakhon Nayok; ^S13^Southern; ^S14^Pathum Thani; ^S15^Nakhon Ratchasima; ^S16^Rayong.

Whole-genome nucleotide alignment using MAFFT identified eight distinct clusters, with cluster 8 being the largest, comprising 43 phages (52.44%) (Figure 1B). Consistent clustering into the same eight groups was independently confirmed using VICTOR, VIRIDIC, and VirClust (Figures S1–S3; Tables S3, S4, and S5), supporting the robustness of the clade assignments.

Within cluster 8 are prophages induced from *B. pseudomallei*, *B. thailandensis*, BTCV, and soil-isolated free phages. This highlights the fluidity of the host for these phages and shows interplay between lysogenic and environmental phages when shaping microbial communities. In addition, MMC-induced *B. pseudomallei* prophages were common and accounted for 46.5% of the cluster, followed by soil-isolated free phages (30.2%). All soil-isolated free phages showed more than 95% average nucleotide identity (ANI) to prophage sequences integrated in *B. pseudomallei* genomes, suggesting that these soil-isolated free phages could have been activated from prophages sequences in the genomes of *B. pseudomallei.*

As depicted in Figure 1B, MMC-induced *B. thailandensis* prophages accounted for only 2.3% of cluster 8 and were found across six of the eight clusters, indicating distinct genomic characteristics and broader diversification compared with *B. pseudomallei* prophages. In contrast, BTCV-induced prophages comprised 20.9% of cluster 8, showing closer genetic relationships with MMC-induced *B. pseudomallei* prophages. The remaining BTCV-induced prophages were distributed across four of the eight clusters.

### Induced *B. pseudomallei* prophages and soil-isolated free phages map to two clades

**Figure 2:**
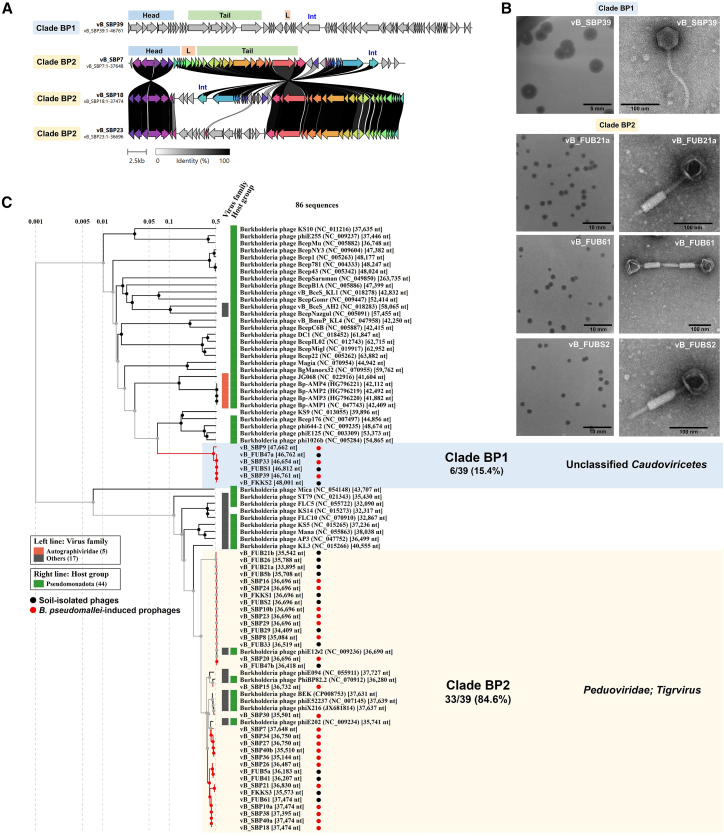
Genomic and morphological characterization of *B. pseudomallei*-associated phages (A) Synteny analysis of representative phages from clades BP1 and BP2. Four conserved genome modules were compared: head, tail, lysis, and accessory modules. Arrows represent ORFs, with colors denoting homologous gene clusters. Gray shading between genomes indicates amino acid identity levels (0%–100%). Clade BP2 differs in genes located downstream of the integrase. (B) Transmission electron micrographs and plaque morphologies of representative phages from each clade. Scale bars, 100 nm and 5–10 mm for TEM and plaque diameters, respectively. Plaque morphologies are shown after multiple rounds of single-plaque purification. (C) Whole-proteome-based phylogenetic tree of 86 *Burkholderia* phages, comprising 39 *B. pseudomallei* phages from this study (red dots*, B. pseudomallei*-induced; black dots, soil-isolated) and 47 *Burkholderia* phages from public databases. Branch colors represent phage source: red, *Burkholderia* phages from this study; black, *Burkholderia* phages from public databases. Colored bars on the left denote predicted viral families (orange, *Autographiviridae*; gray, other families), while colored bars on the right indicate host lineages (green, *Pseudomonadota*). Background shading highlights clades containing both phages from this study and related phages from public databases: blue for clade BP1 and yellow for clade BP2. Unshaded clades consist exclusively of phages from external clades. Percentages indicate the proportion of phages from this study in each clade relative to the total number of *B. pseudomallei*-associated phages from this study.

To better understand the evolution of 23 MMC-induced *B. pseudomallei* prophages, a phylogenetic tree was constructed. Since all 16 soil-isolated free phages showed more than 95% similarity to prophage sequences on *B. pseudomallei* genomes, these free phages were combined in the analysis. According to the whole-genome alignments and gene synteny analysis, the *B. pseudomallei*-associated phages were classified into two main clades: BP1 and BP2 (Table 1). Clade BP2 was the largest, comprising 84.6% (33/39) of the phages, while clade BP1 accounted for 15.4% (6/39). Soil-isolated free phages were distributed across all clades, indicating significant genetic overlap between soil environmental phages and prophages present on the *B. pseudomallei* genomes. Representative plaque morphology of soil-isolated free phages (vB_FUB21a, vB_FUB61, and vB_FUBS2) showed uniformly sized, clear plaques measuring approximately 1.5–2.0 mm in diameter on *B. pseudomallei* UB4 (Figure 2). In contrast, MMC-induced prophage (vB_SBP39) formed larger (∼3.0 mm) and noticeably more turbid plaques under the same assay conditions. No semi-transparent halos surrounding plaques were observed for either free phages or MMC-induced prophage under the assay conditions tested. Thus, plaque appearance differed between free phages and inducible prophages, with free-phage plaques being smaller and clearer on the UB4 host.

Whole-genome alignments using Clinker (Figure 2A) revealed conserved structural regions across clade BP2, with notable genomic variations near the integrase gene (Int), indicating potential hotspots for genetic recombination. Clade BP1 displayed a unique gene composition, lacking shared proteins with clade BP2, suggesting a distinct lineage adapted to specialized niches or alternative hosts.

According to TEM analysis (Figure 2B), all phages exhibited isometric heads but differed in tail morphology: clade BP1 representative had long, non-contractile tails, while clade BP2 representative featured long, contractile tails. Genome-based classification using ICTV-aligned frameworks confirmed that clades BP1 and BP2 belong to the class *Caudoviricetes*. Their placement was supported by three independent analyses, Virus Classification and Tree Building Online Resource (VICTOR) (Table S3), VIRIDIC (Table S4), and VirClust (Table S5), which consistently grouped BP2 with reference members of *Peduoviridae*; Tigrvirus, exhibiting >60% intergenomic similarity and forming a stable genus-level cluster. In contrast, BP1 displayed >70% internal similarity but lacked a closely related reference genome in GenBank, indicating an unclassified lineage within *Caudoviricetes*.

### Comprehensive phylogenetic analysis of *B. pseudomallei* prophages and soil-isolated free phages with public database sequences

**Figure 3:**
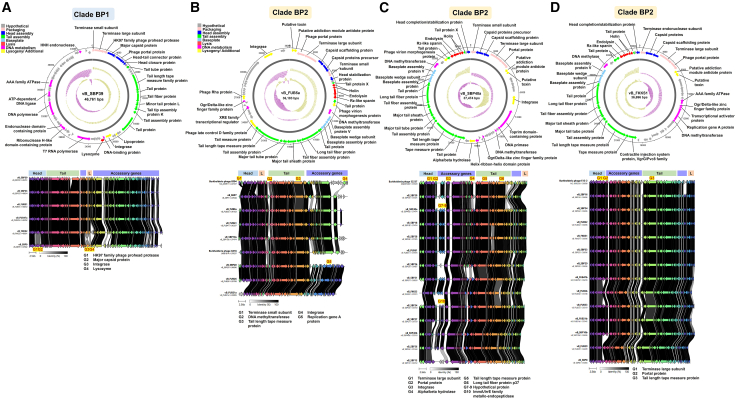
Genomic and synteny analysis of *B. pseudomallei*-associated phage clades (A–D) Representative phages from each clade are shown in (A) BP1 (vB_SBP39), (B) BP2 (vB_FUB5a), (C) BP2 (vB_SBP40a), and (D) BP2 (vB_FKKS1). Upper panels: Genome visualization using DNAplotter shows the phage genome as a dark gray circle organized into eight functional modules (colored bars). The outer scale denotes genome position in bases, with 0 as the origin of replication. The inner circle displays GC skew (yellow: positive; purple: negative), and the outermost circle shows GC content (yellow: above average; purple: below average). Lower panels: Synteny analysis compares four genome modules: head, tail, lysis, and accessory, between phages in each clade and their corresponding reference phages from the GenBank database (clade BP2: phages E094 and X216; clade BP2: phage 52237; clade BP2: phage E12-2). ORFs are shown as arrows, colored by homologous gene clusters, with gray shading indicating amino acid identity (0%–100%). Genes with notable variations among phages within each clade are annotated (highlighted in yellow). Phages in clade BP2 differs in the set of genes located downstream of the integrase.

To contextualize the genomic diversity of *B. pseudomallei* phages, a phylogenetic analysis of 86 sequences comprised of 23 *B. pseudomallei* induced prophages and 16 free phages from this study, along with 47 *Burkholderia* phage sequences from public databases (Table S6) was undertaken. The resulting tree (Figure 2C) showed that previously reported *B. pseudomallei* prophages deposited in public databases clustered within clade BP2, however, clade BP1 was identified as a novel, distinct clade. Phages outside clades BP1-2 were primarily from other *Burkholderia* species, not *B. pseudomallei*. These results suggest that clade BP2 is the predominant cluster for *B. pseudomallei* prophages. Notably, the previously reported *B. pseudomallei* phages, such as AMP1^13^ and ST79,^15^ do not cluster within either clade BP1 or BP2 suggesting that these phages may have distinct evolutionary lineages. To further explore this, genomic organization and synteny analysis were conducted as follows:

Clade BP1: a novel group of *B. pseudomallei* phages. Clade BP1 comprises six phages, representing 15.4% (6/39) of the isolated *B. pseudomallei* phages. Phages in clade BP1 showed minimal similarity to any previously reported phages. The closest match was *Burkholderia* phage phiE125, with only 3.0% coverage (Table 1), indicating a novel group of *Burkholderia* phages. Figure 3A highlights structural genes of clade BP1.

Clade BP2: a common group of *B. pseudomallei* phages. Clade BP2 consists of 33 phages, representing 84.6% (33/39) of all *B. pseudomallei* phages in this study. Publicly available *Burkholderia* phages, such as phiX216 (JX681814) and phiE202 (NC_009234), also cluster within BP2, indicating conserved genomic features (Table 1), suggesting close genetic relationships.

Structural and replication genes were identified and illustrated in the circular genome maps (Figures 3B–3D). A key genomic difference from clade BP1 is the presence of putative toxin-antitoxin system (TAS)-like genes in clade BP2. These genes were identified based on their 100% amino acid sequence identity of encoded proteins to previously reported TAS components, including a RelE/ParE family toxin (e.g., MBK3339294.1, *B. pseudomallei*; AJX76198.1, *Burkholderia* phage phiE094; QWY84944.1, *Burkholderia* phage PK23) and a probable antitoxin homologous to addiction module antidote proteins (e.g., ACQ98486.1, *B. pseudomallei* MSHR346; QWY84945.1, *Burkholderia* phage PK23). Functional domain prediction using InterPro classified these ORFs within the IPR014056 (RelE/ParE-like toxins) and IPR014057 (antitoxin-like proteins) families. Structural homology analysis via HHpred further supported these annotations, revealing high-confidence matches to the HigB toxin (PDB: 6AF4_C, probability = 99.4%) and the putative antitoxin HigA3 (PDB: 6LTZ_A, probability = 95.3%) (Table S7). Furthermore, these phages harbor four lysis-associated genes, compared to one in clade BP1, suggesting enhanced lysis mechanisms for environmental adaptability. Synteny analysis (Figures 3B–3D) highlights conserved structural and replication genes across clade BP2, indicating shared origins.

### *B. thailandensis* and BTCV prophages were grouped into seven clades

**Figure 4:**
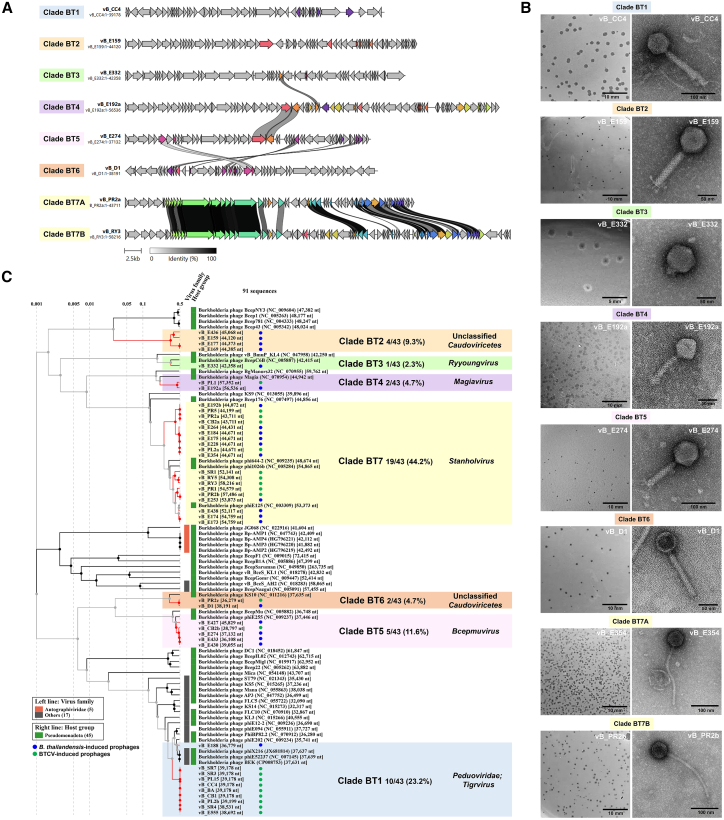
Genomic and morphological characterization of MMC-induced *B. thailandensis*- and BTCV phages (A) Synteny analysis of representative phages from clades BT1-BT7. Four conserved genome modules are compared: head, tail, lysis, and accessory modules. Arrows represent ORFs, with colors denoting homologous gene clusters. Gray shading between genomes indicates amino acid identity levels (0%–100%). (B) Transmission electron micrographs and plaque morphologies of representative phages from each clade (BT1-BT7). Scale bars, 50–100 nm and 5–10 mm for TEM and plaque diameters, respectively. Plaque morphologies are shown after multiple rounds of single-plaque purification. (C) Whole-proteome-based phylogenetic tree of 91 *Burkholderia* phages, comprising 43 *B. thailandensis*/BTCV-associated phages from this study (blue dots, *B. thailandensis*-induced; green dots, BTCV-induced) and 48 *Burkholderia* phages from public databases. Branch colors represent phage source: red, *Burkholderia* phages from this study; black, *Burkholderia* phages from public databases. Colored bars on the left denote predicted viral families (orange, *Autographiviridae*; gray, other families), while colored bars on the right indicate host lineages (green, *Pseudomonadota*). Background shading highlights clades containing both phages from this study and related phages from public databases: BT1 (blue), BT2 (orange), BT3 (green), BT4 (purple), BT5 (pink), BT6 (dark orange), and BT7 (yellow). Unshaded clades consist exclusively of phages from external clades. Percentages indicate the proportion of phages from this study in each clade relative to the total number of *B. thailandensis*-associated phages from this study.

Total 43 prophage genome sequences induced from *B. thailandensis* and BTCV were also investigated. Phage classification into seven distinct clades (BT1-BT7) was primarily based on genome-wide alignment and synteny patterns (Table 1). Clade BT1 (10 phages) and clade BT7 (19 phages) were the largest, comprising 23.3% and 44.2%, respectively. BTCV-induced prophages were predominantly concentrated in clades BT1 and BT7, whereas *B. thailandensis*-induced phages were distributed across all seven clades, indicating greater genomic diversity. Whole-genome alignments (Figure 4A) revealed significant differences among the seven phage clades, suggesting distinct evolutionary pathways and functional specializations that reflect the adaptability and diverse ecological roles of *B. thailandensis* and BTCV phages.

Following plaque assays using the corresponding *Burkholderia* host strains listed in Tables 2 and S1, MMC-induced prophages from *B. thailandensis* and BTCV produced plaques with distinct but partially overlapping morphologies (Figure 4B). Plaques formed by the BTCV phage vB_CC4 (clade BT1) were turbid, with diameters of approximately 1.5–2.0 mm. In contrast, plaques formed by *B. thailandensis* phages vB_E159 (clade BT2) and vB_E274 (clade BT5) were small, clear pinpoint plaques, generally less than 1.0 mm in diameter. Notably, *B. thailandensis* phage vB_E332 (clade BT3) formed turbid plaques of approximately 2.0 mm in diameter with diffuse margins, giving a halo-like appearance. Meanwhile, *B. thailandensis* phage vB_E192a (clade BT4) generated markedly turbid plaques with poorly defined edges, typically ranging from 1.0 to 2.0 mm in diameter. In comparison, *B. thailandensis* phage vB_D1 (clade BT6) and BTCV phage vB_PR2b (clade BT7) consistently formed clear plaques of approximately 1.0 mm in diameter. Overall, none of the MMC-induced *B. thailandensis* and BTCV phages produced true halo plaques under the assay conditions, with the exception of phage vB_E332 (clade BT3), which exhibited a halo-like appearance.

**Table 2:** Bacterial strains used in this study

Bacteria	Numbers of isolates	Isolates	Sources	Reference
*B. pseudomallei*	41	K96243	Clinical	Wuthiekanun et al.^19^
		UB1, UB2, UB3, UB4, UB5, UB6, UB7, UB8, UB9, UB10, UB11, UB12, UB13, UB14, UB15, UB16, UB17, UB18, UB19, UB20, UB21, UB22, UB23, UB24, UB25, UB26, UB27, UB28, UB29, UB30, UB31, UB32, UB33, UB34, UB35, UB36, UB37, UB38, UB39, UB40	Soil	This study
Typical *B. thailandensis*	40	DW503	Soil	Burtnick et al.^20^
		D1	Soil	Jitprasutwit et al.^21^
		E152, E153, E154, E158, E159, E169, E173, E174, E175, E177, E184, E188, E192, E201, E202, E205, E207, E228, E234, E246, E253, E264, E27, E274, E332, E352, E354, E360, E421, E426, E427, E430, E433, E435, E436, E438, E440, E441	Soil	Hantrakun et al.^6^
*B. thailandensis* Capsule Variant (BTCV)	20	E555	Soil	Sim et al.^5^
		SBXCB001, SBXCB002, SBXCC001, SBXCC003, SBXCC004, SBXPL001, SBXPL002, SBXPL003, SBXPL015, SBXPR001, SBXPR002, SBXPR005, SBXRY003, SBXRY005, SBXSR001, SBXSR003, SBXSR004, SBXSR007	Soil	Hantrakun et al.^6^
		WBXUBA33005104	Water	Hantrakun et al.^6^

TEM analysis (Figure 4B) showed that although all MMC-induced *B. thailandensis* and BTCV prophages exhibited isometric heads, they displayed diverse tail morphologies. Clades BT1, BT2, and BT5 had long, contractile tails; BT4 and BT6 also appeared to have long tails, although the contractile sheath was not clearly visible in the micrographs; BT3 had short tails; and BT7 had long, non-contractile tails. Genome-based classification using ICTV-aligned analyses (VIRIDIC, VICTOR, and VirClust) further resolved these clades within the class *Caudoviricetes*. BT5 showed >90% intergenomic similarity to reference *Peduoviridae*; Bcepmuvirus genomes (e.g., phiE255), consistent with placement in this genus. BT3 clustered with members of Ryyoungvirus, whereas BT4 shared ∼60% similarity with Magiavirus. BT7 (including phiE125 and phiE264-1) formed a coherent group affiliated with Stanholtvirus. In contrast, BT1, BT2, and BT6 exhibited internal similarity >70% but lacked close matches to established genera, indicating unclassified lineages within *Caudoviricetes*.

### Comprehensive phylogenetic analysis of *B. thailandensis* and BTCV prophages across clades and public databases

The phylogenetic analysis of 91 sequences, including 43 *B. thailandensis* and BTCV prophages identified in this study, along with 48 public *Burkholderia* phages sequences (Table S6), revealed diversity extending beyond the seven major clades (BT1-BT7). As illustrated in Figure 4C, most *B. thailandensis* and BTCV phages clustered within BT1-BT7, while phages from other *Burkholderia* species were distributed outside these clades. Clade BT1 aligns with *B. pseudomallei* phage phiX216, while BT2 shows no close similarity to known *Burkholderia* phages. Clades BT3 and BT6 align with *B. cepacia* complex (BCC) phages BcepC6B and KS10, respectively, while clade BT4 resembles *B. cenocepacia* phage Magia. Clades BT5 and BT7 include phages like *B. thailandensis* phiE255 and phiE125, respectively. These results suggest shared origins and conserved elements between *B. thailandensis*, *B. pseudomallei*, and BCC phages. The genomic organization and synteny of *B. thailandensis* and BTCV phages were comparatively analyzed within clades as follows:

Clade BT1: the only BT clade showing similarity to *B. pseudomallei* phages. Clade BT1 comprises 10 phages (Table 1), representing 23.3% (10/43) of *B. thailandensis* and BTCV phages. Genome mapping and synteny (Figure S4) resemble *B. pseudomallei* clade BP2 (Figures 3B–3D), and BLAST analysis shows 79%–91% coverage and 93.5%–98.3% identity with phage phiX216 (Table 1), suggesting a shared ancestor. Putative toxin-antitoxin systems (TAS) were identified in clade BT1 phages, showing 100% coverage and 100% nucleotide identity to TAS sequences found in *B. pseudomallei* clade BP2, suggesting they may encode homologous functional modules. Of the 10 phages in clade BT1, nine are from BTCV and only one from *B. thailandensis*, suggesting that BTCV may act as a reservoir for *B. pseudomallei*-like phages that can be transferred to other *Burkholderia* species, such as soil-dwelling *B. thailandensis*, which often coexists with BTCV and facilitates genetic exchange in the same environments.

**Figure 5:**
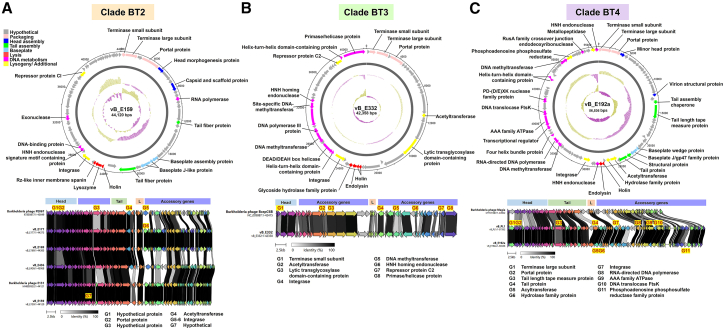
Genomic and synteny analysis of phages in *B. thailandensis* clades BT2, BT3, and BT4 (A–C) Representative phages from each clade are shown in (A) BT2 (vB_E159), (B) BT3 (vB_E332), and (C) BT4 (vB_E192a). Upper panels: Genome visualization using DNAplotter shows the phage genome as a dark gray circle organized into eight functional modules (colored bars). The outer scale denotes genome position in bases, with 0 as the origin of replication. The inner circle displays GC skew (yellow: positive; purple: negative), and the outermost circle shows GC content (yellow: above average; purple: below average). Lower panels: Synteny analysis compares four genome modules: head, tail, lysis, and accessory, between phages in each clade and their corresponding reference phages from the GenBank database (Clade BT2: phages PE067 and E131; Clade BT3: phage BcepC6B; Clade BT4: phage Magia). ORFs are shown as arrows, colored by homologous gene clusters, with gray shading indicating amino acid identity (0%–100%). Genes with notable variations among phages within each clade are annotated (highlighted in yellow).

Clade BT2 comprised four *B. thailandensis*-derived phages (vB_E159, vB_E169, vB_E177, and vB_E436), representing 9.3% of the 43 *B. thailandensis* and BTCV prophages (Table 1). Phages in this clade shared 73%–75% coverage and 96.5%–98.6% nucleotide identity with *B. thailandensis* phage PE067, while one phage (vB_E159) showed complete coverage and identity to phage E131 (Table 1). Synteny analysis (Figure 5A) revealed conserved structural gene organization within the clade. Across BT2 phages, conserved accessory genes with annotations included a CI-type repressor and a site-specific integrase, both features commonly associated with lysogeny regulation.

Clade BT3 consisted of a single phage (vB_E332) derived from MMC-induced *B. thailandensis* E332, representing 2.3% (1/43) of all *B. thailandensis* and BTCV phages (Table 1). It shared 75% genome coverage and 86.2% nucleotide identity with *B. cepacia* complex phage BcepC6B (Table 1). Phage vB_E332 (Figure 5B) was found to possess a gene encoding a putative lytic transglycosylase with 91.9%–95.5% amino acid sequence identity to annotated proteins from *Burkholderia* (e.g., WP_119337678.1) and phage homologs (e.g., YP_009800740.1). Domain analysis identified a conserved soluble lytic transglycosylase (SLT) domain (Pfam: PF01464). Structural prediction by HHpred supported this classification, showing 99.6% similarity to the lytic murein transglycosylase from *Pseudomonas aeruginosa* (PDB: 5OHU_A) (Table S7). Synteny analysis (Figure 5B) comparing vB_E332 with BcepC6B revealed conserved gene organization, with regional variations that likely reflect evolutionary divergence between the two phages.

Clade BT4 consisted of two phages, vB_E192a derived from *B. thailandensis* and vB_PL1 derived from BTCV, representing 4.7% (2/43) of all *B. thailandensis* and BTCV phages (Table 1). They shared 40%–42% genome coverage and 90.1%–90.2% nucleotide identity with *B. cenocepacia* phage Magia (Table 1). Phage vB_E192a (Figure 5C) contained several annotated accessory genes showing high amino acid sequence identity (98.1%–100%) to previously reported *Burkholderia* proteins. These included a putative phosphoadenosine phosphosulfate (PAPS) reductase (98.1% identity to WP_180986649.1) containing the conserved PF01507 domain; a putative metallopeptidase (99.5% identity to WP_043296378.1) with a PF18894 domain; and a putative acyltransferase (100% identity to WP_158338970.1) classified within the acyltransferase 3 family (IPR050879). HHpred predictions further supported similarities to known protein structures (Table S7). Synteny analysis (Figure 5C) revealed partial conservation of structural genes with phage Magia, while variation in accessory and replication-associated genes reflect evolutionary divergence within the clade.

Across clades BT2-BT4, a relatively low proportion of genes encoded known structural proteins, whereas a high proportion corresponded to hypothetical proteins: 67.2% (45/67 ORFs) in BT2, 61.5% (32/52 ORFs) in BT3, and 64.2% (52/81 ORFs) in BT4 (Figures 5A–5C). These observations highlighted the presence of numerous uncharacterized ORFs, although their functional roles remained undetermined and will require future experimental investigation.

**Figure 6:**
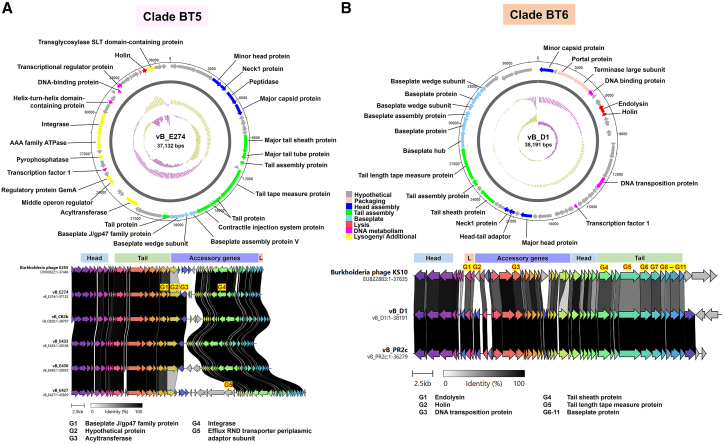
Genomic and synteny analysis of phages in *B. thailandensis* clades BT5 and BT6 (A and B) Representative phages from each clade are shown in (A) BT5 (vB_E274) and (B) BT6 (vB_D1). Upper panels: Genome visualization shows the phage genome as a dark gray circle organized into eight functional modules (colored bars). The outer scale denotes genome position in bases, with 0 as the origin of replication. The inner circle displays GC skew (yellow: positive; purple: negative), and the outermost circle shows GC content (yellow: above average; purple: below average). Lower panels: Synteny analysis compares four genome modules: head, tail, lysis, and accessory, between phages in each clade and their corresponding reference phages from the GenBank database (clade BT5: phage E255; clade BT6: phage KS10). ORFs are shown as arrows, colored by homologous gene clusters, with gray shading indicating amino acid identity (0%–100%). Genes with notable variations among phages within each clade are annotated (highlighted in yellow).

Clade BT5 consisted of five phages (Table 1), one derived from MMC-induced BTCV and the others from *B. thailandensis*, representing 11.6% (5/43) of all *B. thailandensis* and BTCV phages. Phages in this clade shared 80%–91% genome coverage and 95.5%–100% nucleotide identity with *B. thailandensis* phage phiE255 (Table 1). Phage vB_E274 (Figure 6A) contained annotated regulatory genes showing 100% amino acid identity to previously reported proteins in *Burkholderia* phages and members of the *pseudomallei* group. These included a putative GemA-like protein (WP_015985046.1) belonging to the GemA family (IPR009363; Pfam: PF06252), and a middle operon regulator (MOR; WP_015985055.1) possessing the PF08765 domain. HHpred analyses further supported structural similarity to known proteins (Table S7). Synteny analysis (Figure 6A) showed high conservation with *B. thailandensis* phage phiE255, reflecting shared origins. Additional genes detected in clade BT5 phages, including an acetyltransferase and an efflux RND transporter periplasmic adaptor, reflected genetic variation within the clade, although their functional relevance remains undetermined.

Clade BT6 consisted of two phages, vB_D1 derived from *B. thailandensis* and vB_PR2c derived from BTCV, representing 4.6% (2/43) of all *B. thailandensis* and BTCV phages (Table 1). These phages shared 70%–74% genome coverage and 83.3% nucleotide identity with *B. cepacia* complex phage KS10 (Table 1). Interestingly, no annotated accessory or lysogeny-associated genes were identified in phage vB_D1. Synteny analysis (Figure 6B) revealed partial gene conservation between *B. cepacia* complex phage KS10 and *B. thailandensis* phage vB_D1, indicating shared evolutionary origins.

Clade BT7: the most common *B. thailandensis* phage group. Clade BT7 comprised 19 phages (Table 1), representing 44.2% (19/43) of all *B. thailandensis* and BTCV phages, making it the largest group. It included 9 phages from BTCV and the rest from *B. thailandensis*. Phages in clade BT7A shared 86%–100% coverage and 95.7%–100% nucleotide identity with *B. thailandensis* phage phiE264.1,^11^ while clade BT7B shared 64%–97% coverage and 95.1%–99.9% identity with phage phiE125 (Table 1). Synteny analysis (Figure S5) revealed strong conservation of structural gene regions across clades BT7A and BT7B, indicating shared origins, while showing variability in accessory genes. Regulatory genes, including CII and CII repressors, were identified in vB_PR2a, a BTCV prophage in clade BT7A (Figure S5). Phage vB_RY3 (Figure S5) was found to encode two putative accessory genes with proteins showing 100% amino acid identity to known *Burkholderia* proteins, including a putative RelE-like toxin (WP_004549735.1), classified under the RelE/ParE toxin family (IPR052747; Pfam: PF05016) and supported by HHpred structural similarity to the RelE toxin from *Escherichia coli* (PDB: 2KHE_A, 99.5% probability). A putative phosphoadenosine phosphosulfate (PAPS) reductase (WP_009896489.1), containing conserved PF01507 and IPR050128 domains, was also identified, with HHpred predicting structural similarity to the PAPS reductase from *Vibrio vulnificus* (PDB: 6VPU_C, 99.9% probability) (Table S7). The specific biological roles of these genes within the prophage context remain to be experimentally verified.

## Discussion

In this study, we analyzed the diversity of prophages induced from selected strains of *B. pseudomallei*, *B. thailandensis*, and BTCV, together with *Burkholderia* phages isolated from soils in the northeast of Thailand. Soil sampling was conducted at two different sites, one from the province of Khon Kaen and one from Ubon Ratchathani. Although *B. pseudomallei* and culturable free phages were recovered from soil collected in Ubon Ratchathani province, *B. pseudomallei* was not cultured from any of the 50 soil samples collected in Khon Kaen province. However, culturable *Burkholderia* phages were detected in samples from both provinces. This discrepancy may be attributed to several factors, such as differences in environmental conditions and nutrient-depleting agricultural practices that may impact the viability and distribution of both bacteria and phages in the soil.^22^^,^^23^ In addition, the location of soil sampling in Ubon Ratchathani province had been identified (personal communication) previously as a hot spot of *B. pseudomallei* whereas the sampling site at Khon Kaen province was randomly selected and not known as an abundant source of such bacteria. Notably, we cannot rule out the possibility that the positive phage/negative *B. pseudomallei* isolation may result from several reasons, such as (1) prophages being activated from *B. pseudomallei* genome, leading to bacterial lysis and thus the bacteria could not be cultured, or (2) the culturable free phages may have *B. thailandensis* or other, yet unknown, bacterial species as their bacterial host.

The geographical distribution analysis of phages isolated from soil showed that phages identified in both provinces belong to the clades BP1 (a novel group that did not show homology to any other phages deposited into the GenBank database) and BP2, which represent phages commonly found across multiple *B. pseudomallei* isolates. This finding indicates that no significant differences were found in terms of phage clade distribution between the two endemic regions of *B. pseudomallei*. The consistency of phage clade presence across these areas suggests a stable and persistent phage population, supporting their potential use as indicators for monitoring *B. pseudomallei* spread in the future. This suggestion is supported by previous reports that environmental phage surveillance is useful for tracking *Salmonella typhi* in endemic settings.^24^ However, further investigation with additional sampling sites should be undertaken to more extensively investigate this in conjunction with the bacterial detection data and environmental context.

Pertinent to the 82 MMC-induced *Burkholderia* phages and soil-isolated free phages, intergenomic clustering identified eight distinct clusters, with cluster 8 being the largest (52.44%). Cluster 8 was composed of prophages induced from *B. pseudomallei*, *B. thailandensis*, and BTCV, as well as soil-isolated free phages, indicating the interplay between lysogenic and environmental phages in shaping microbial communities. Of note, this study identified for the first time that all soil-isolated free phages showed more than 95% average nucleotide identity to prophage sequences in *B. pseudomallei* genomes, indicating that these soil-dwelling phages may have originated from *B. pseudomallei* genomes. However, the induction factors in the soil environment that contribute to prophage induction require further investigation, particularly as environmental cues have been shown to trigger prophage induction in soil bacteria.^25^

The phylogenetic tree constructed from 39 *B. pseudomallei* phages, including MMC-induced prophages (23 phages) and free phages directly isolated from soil (16 phages) revealed that these phages could be classified into two clades: BP1 and BP2. Nucleotide BLAST analysis revealed that phages in clade BP1 are a novel group of *Burkholderia* phages that have not been reported before, supporting the notion that *Burkholderia* prophages diversity exceeds current *in silico* predictions.

The identification of putative toxin-antitoxin system (TAS)-like gene pairs in phage clade BP2 raises interesting questions regarding their potential biological roles. Although predicted products of these genes share 100% amino acid sequence identity with previously reported TAS components from *Burkholderia* genomes and phages, and exhibit conserved domain signatures, their actual functions within the phage context remain unresolved. TAS are genetic modules typically composed of a stable toxin and a labile antitoxin. They were originally described in the context of plasmid maintenance via post-segregational killing (or “plasmid addiction”).^26^ However, TAS are now recognized to play broader roles beyond plasmid stability, including contributing to phage stability,^27^ regulating lysogeny,^28^ and modulating bacterial stress responses,^29^ as has been observed in other phage-host systems. However, without experimental validation, their roles in enhancing host adaptability remain speculative. Further functional studies, such as gene expression profiling or mutational analysis, are needed to determine whether these modules operate as bona fide TAS or serve distinct phage-specific functions.

Interestingly, we found from this study that MMC-induced *B. pseudomallei* prophages in clade BP2 share similarities (>97% ANI) with phage vB_HM387 which was isolated from the hemoculture of melioidosis patients^17^ indicating that prophages were not only induced and detected *in vitro* but also in a clinical sample from a melioidosis patient. The factor(s) in the blood of melioidosis patients that trigger release of prophage particles from bacteria and mediate their appearance in the blood are largely unknown. Consequently, the impact of phage presence *in vivo* on the pathogenesis or severity of melioidosis requires further investigation.

Strikingly, none of the culturable free phages and induced prophages of *B. pseudomallei* collected from soil in this study carried a gene encoding the virulence-associated protein E (*vapE*) that was previously reported in the genome of *B. pseudomallei* phage vB_HM795 collected from the hemoculture of melioidosis patient.^17^ Another key observation in this study is that the previously reported *Burkholderia* phages isolated from northeast of Thailand including ST79,^15^ and AMP1-like phages^13^ were not found in this study. The possible explanation may be due to several reasons such as differences of *B. pseudomallei* host strains and the locations of soil sampling. Anyhow, this indicates that diversity of *B. pseudomallei* is present in the environment.

In comparison with *B. pseudomallei* phages, 43 MMC-induced *B. thailandensis* and BTCV prophages were analyzed and could be classified into seven clades (BT1-BT7). This is the first study to report that, under our experimental conditions, BTCV displayed a higher proportion of MMC-inducible prophages than *B. pseudomallei* or *B. thailandensis*. BTCV-induced prophages were concentrated in clades BT1 and BT7, whereas *B. thailandensis*-induced phages were distributed across all seven clades. Clade BT7 is the most common phage group in *B. thailandensis*. Only clade BT1 showed high sequence similarity to *B. pseudomallei* clade BP2.

Plaque morphology analysis showed that most *B. pseudomallei* phages in this study produced clear or turbid plaques without detectable halo formation under the conditions tested, consistent with previously described *B. pseudomallei* phages such as AMP1,^13^ ST79,^15^ and C34,^30^ which also form clear plaques without halos. In contrast, the *B. pseudomallei* phage vB_BpP_HN01^31^ has previously been reported to develop a pronounced halo after extended incubation, a feature associated with capsular polysaccharide-degrading activity.^32^ The lack of halo formation in our *B. pseudomallei* phages may reflect the absence or limited activity of capsule-degrading enzymes, as no clear homologs of known depolymerases or tail spike-associated hydrolases were identified in genomes of our isolated *Burkholderia* phages. However, enzyme activity may be condition-dependent and influenced by the host strain used for plaque assays, and therefore cannot be entirely excluded.

For *B. thailandensis* and BTCV, plaque morphologies were more variable, ranging from clear pinpoint plaques to larger turbid plaques. Notably, the *Burkholderia* phage vB_E332 in clade BT3 exhibited a halo-like appearance. This distinctive phenotype may be associated with the presence of a predicted glycoside hydrolase family protein encoded in its genome (Figure 5B). Enzymes of the glycoside hydrolase family have been reported to cleave glycosidic bonds in bacterial capsular components, a function that has been associated with halo formation.^32^ However, functional validation is required to confirm the role of glycoside hydrolase activity in generating the halo-like appearance observed for phage vB_E332.

In *B. thailandensis* phages, several annotated accessory genes were identified across multiple clades. In clade BT4, a gene encoding a putative lytic transglycosylase was detected, showing similarity to enzymes involved in peptidoglycan-associated processes. Additional annotated genes in this clade included those encoding phosphoadenosine phosphosulfate (PAPS) reductase, metallopeptidase, and acyltransferase, each containing conserved domains characteristic of their respective protein families. Regulatory genes such as *gemA* and *mor* were also identified. Although these genes exhibit conserved domain signatures and high sequence similarity to known proteins, their specific roles in the *B. thailandensis* phage context remain unknown and will require further experimental investigation.

This study showed that both *B. pseudomallei* phage clades and four out of seven *B. thailandensis* phage clades (BT1, BT2, BT5, and BT7) were identified in Ubon Ratchathani province, an area of high endemicity for *B. pseudomallei*. The high phage diversity observed in Ubon Ratchathani, particularly the presence of multiple *B. thailandensis* and *B. pseudomallei* phage clades, indicates a complex and dynamic phage-bacteria interaction in this area. Potential ecological interactions between prophages and bacteria may facilitate gene flow between these closely related *Burkholderia* species, although the underlying mechanisms and frequency of such events remain unclear. Observations in other bacterial systems show that mobile genetic elements can mediate cross-species gene transfer, but whether similar processes occur in *Burkholderia* requires further investigation. For examples, *B. thailandensis* phages clades BT3 BT4 and BT6 share genomic links with *B. cepacia* complex (BCC) and *B. cenocepacia* phages, highlighting their connections and potential for cross-species genetic exchange. A similar phenomenon has been seen in *Vibrio cholerae*, where *V. cholerae* El Tor acquired the classical CTX prophage from *V. cholerae* O141 in chitin-rich aquatic environments,^33^ highlighting how prophages can mediate gene transfer across strains in nature. Likewise, in *Streptococcus pyogenes*, prophages carrying toxin genes can integrate into diverse strains, contributing to the spread of virulence factors, antibiotic resistance, and shaping bacterial population dynamics.^34^

In conclusion, *B. pseudomallei*, *B. thailandensis*, and BTCV carried prophages on their genomes that could be induced with MMC to generate different culturable phage particles for characterization. Whole-genome sequencing and genome analysis indicated that the induced *Burkholderia* prophages are diverse with some phages shared among *B. pseudomallei*, *B. thailandensis*, and BTCV. *B. pseudomallei* prophage clade BP1 is a novel group which that has not been reported before, whereas clade BP2 is similar to previously identified phages. Phylogenomic analysis revealed strong genetic similarity between *B. thailandensis* and *B. pseudomallei* prophages, reflecting their close evolutionary relatedness. The wide distribution of *B. thailandensis* phages suggests substantial ecological diversity. BTCV phages exhibited genomic similarity to prophages from both species, consistent with their intermediate phylogenomic position. While these patterns may indicate broader genomic connectivity among *Burkholderia* lineages, the underlying mechanisms and biological relevance remain unclear and will require future functional investigation.

### Limitations of the study

The number of phages recovered in this study was influenced by both environmental and methodological factors. Soil samples were collected from only two melioidosis-endemic sites, where isolation outcomes may vary with seasonal conditions, soil composition, and agricultural practices. Importantly, this work focused specifically on culturable, MMC-inducible prophages and therefore did not capture non-inducible or defective elements. Because whole-genome sequences of most host strains, including all BTCV isolates, were unavailable, *in silico* prophage prediction could not be carried out. As a result, the isolation frequencies presented here reflect only prophages detectable under the MMC-induction conditions used.

## Resource availability

### Lead contact

Requests for further information and resources should be directed to and will be fulfilled by the lead contact, Dr. Sunee Korbsrisate (sunee.kor@mahidol.ac.th).

### Materials availability

This study did not generate new unique reagents.

### Data and code availability

**Data:** Phage genome sequences have been deposited in the NCBI database, with accession numbers; vB_SBP9: PQ789808; vB_SBP39: PV072743; vB_FUBS1: PV072773; vB_FKKS2: PV072744; vB_SBP33: PV072774; vB_FUB47a: PV072775; vB_SBP20: PQ789809; vB_FUB26: PV987627; vB_FUB21a: PV072745; vB_SBP7: PV111787; vB_SBP10a: PV976088; vB_FUB5a: PQ789810; vB_FUB41: PV987629; vB_SBP30: PQ789829; vB_SBP26: PV976089; vB_FUB61: PV072746; vB_SBP38: PV987637; vB_SBP40a: PQ789811; vB_SBP18: PV987632; vB_SBP15: PV111788; vB_SBP21: PV111789; vB_FKKS3: PV976091; vB_SBP36: PV111790; vB_SBP40b: PV987638; vB_SBP34: PQ789812; vB_SBP27: PV987635; vB_FUB21b: PV976092; vB_FUB47b: PV987630; vB_FKKS1: PQ789813; vB_SBP10b: PV987631; vB_SBP23: PV987633; vB_FUB29: PV976093; vB_FUBS2: PV987626; vB_SBP24: PV987634; vB_SBP8: PV976090; vB_SBP29: PV987636; vB_FUB5b: PV976094; vB_FUB33: PV987628; vB_SBP16: PV072747; vB_E188: PQ789830; vB_SR4: PV072748; vB_E555: PQ789814; vB_PL2b: PV072749; vB_SR7: PV072750; vB_SR3: PV072751; vB_PL15: PV072752; vB_CC4: PV072753; vB_CB1: PQ789815; vB_BA: PV072754; vB_E159: PQ789816; vB_E436: PQ789831; vB_E177: PQ789817; vB_E169: PV072755; vB_E332: PQ789818; vB_PL1: PV072756; vB_E192a: PQ789819; vB_E427: PQ789820; vB_CB2b: PV072757; vB_E430: PV072758; vB_E433: PV072759; vB_E274: PQ789821; vB_D1: PQ789822; vB_PR2c: PV072760; vB_PR5: PV072761; vB_E192b: PV072762; vB_PR2a: PQ789823; vB_CB2a: PV072763; vB_E264: PV072772; vB_E175: PV072769; vB_E184: PV072770; vB_E228: PV072771; vB_PL2a: PV072768; vB_E354: PQ789824; vB_SR1: PQ789825; vB_RY5: PV072764; vB_RY3: PQ789826; vB_PR1: PV072765; vB_PR2b: PQ789827; vB_E253: PV072766; vB_E438: PQ789832; vB_E174: PQ789828; vB_E173: PV072767. All data are publicly available as of the date of publication.

**Code:** This paper does not report original code.

**Additional information:** Any additional information required to reanalyze the data reported in this paper is available from the lead contact upon request.

## Acknowledgments

This project is funded by the National Research Council of Thailand (10.13039/501100004704NRCT) and 10.13039/501100004156Mahidol University (N42A660430) and the Royal Society NMG\R1\191196 - Newton Mobility Grants 2019. V.W. is supported by The Wellcome Trust of Great Britain (grant no. 089275/Z/09/Z). P.W., V.M., S.J., and S.K. are supported by the 10.13039/501100013238Faculty of Medicine Siriraj Hospital, Mahidol University. Finally, we acknowledge the constructive suggestions from Dr. Prasert Auewarakul, Faculty of Medicine Siriraj Hospital, Mahidol University.

## Author contributions

Conceptualization, P.W. and S.K.; methodology, P.W. and S.K.; investigation, P.W., V.M., S.J., P.A., D.J.B., and O.G.; writing-original draft, P.W. and S.K.; writing-review & editing, P.W., V.W., M.R.J.C., E.E.G., O.G., and S.K.; funding acquisition, P.W., O.G., and S.K.; resources, V.W. and S.C.; supervision, O.G, and S.K.

## Declaration of interests

The authors declare no competing interests.

## STAR★Methods

### Key resources table

**Table:** 

REAGENT or RESOURCE	SOURCE	IDENTIFIER
**Bacterial and virus strains**		

All bacterial strain	See Table 2	N/A
Phages	This study	See Tables 1 and S1

**Chemicals, peptides, and recombinant proteins**		

Luria-Bertani broth	Criterion™, Hardy Diagnostics, USA	Cat# C9271
Agar powder	TM MEDIA®	REF#1201
Sodium chloride (NaCl)	VWR Chemicals BDH®	CAS# 7647-14-5
Calcium Chloride Dihydrate	Vivantis Technologies	CAS# 10035-04-8
Magnesium Sulfate Heptahydrate (MgSO4 · 7H2O)	VWR Chemicals BDH®	CAS# 10034-99-8
Mitomycin C	Sigma Aldrich, USA	Cat# 50-07-7
Formvar/carbon-coated copper grids (200 mesh)	Sigma Aldrich, USA	Cat# TEM-FCF200CU50
DNase I	Thermo Fisher Scientific	Cat# EN0521
RNase A	Thermo Fisher Scientific	Cat# EN0531
Proteinase K	Thermo Fisher Scientific	Cat# EN0491
Tris	Vivantis Technologies	Cat# PR0612
Ethylenediaminetetraacetic acid (EDTA)	Sigma Aldrich, USA	Cat# E5134
Sodium dodecyl sulfate (SDS)	Sigma Aldrich, USA	Cat# L3771
Phenol	Thermo Fisher Scientific	CAS# 108-95-2
Chloroform	Thermo Fisher Scientific	CAS# 67-66-3
Isoamyl alcohol	Fisher Chemical™	Cat# BPA3934
Ethanol	VWR Chemicals BDH®	Cat# VWR20821
Sodium acetate	Sigma Aldrich, USA	Cat# S8750

**Critical commercial assays**		

Illumina DNA Library Prep kit	Illumina, Inc., USA	https://www.illumina.com/products/by-type/sequencing-kits/library-prep-kits/illumina-dna-prep.html
Latex agglutination test	Duval et al.^35^	https://www.tm.mahidol.ac.th/micro-immuno/portfolio-item/latex/

**Deposited data**		

Whole genome sequences of phages	This paper	GenBank accession numbers see Table S1

**Software and algorithms**		

Sickle v1.33	Joshi et al.^36^	https://github.com/najoshi/sickle; RRID: SCR_006800
Trimmomatic v0.39	Bolger et al.^37^	usadellab.org; RRID: SCR_011848
SPAdes v3.6.0	Bankevich et al.^38^	https://github.com/ablab/spades; RRID: SCR_000131
Unicycler v0.4.8	Wick et al.^39^	https://github.com/rrwick/Unicycler; RRID: SCR_024380
Prokka v1.12	Seemann^40^	https://github.com/tseemann/prokka; RRID: SCR_014732
BLASTP	NCBI	https://blast.ncbi.nlm.nih.gov/Blast.cgi
PhageTerm v1.0.12	Garneau et al.^41^	https://sourceforge.net/projects/phageterm/
HHPred v2.08	Zimmermann et al.^42^	https://toolkit.tuebingen.mpg.de/tools/hhpred; RRID: SCR_010276
InterPro v106.0	Blum et al.^43^	https://www.ebi.ac.uk/interpro/; RRID: SCR_006695
Artemis v17.0.1	Rutherford et al.^44^	https://github.com/sanger-pathogens/Artemis; RRID: SCR_004267
MAFFT v7.505	Katoh et al.^45^	https://mafft.cbrc.jp/alignment/server/index.html; RRID: SCR_011811
Tree of Life (iTOL) v6.5.8	Letunic et al.^46^	https://itol.embl.de/
Clinker v0.0.25	Gilchrist et al.^47^	https://github.com/gamcil/clinker; RRID: SCR_016140
VipTree v1.1.3	Nishimura et al.^48^	https://github.com/yosuken/ViPTreeGen
VICTOR v1	Meier-Kolthoff et al.^49^	https://ggdc.dsmz.de/victor.php
VIRIDIC v1.1	Moraru et al.^50^	https://rhea.icbm.uni-oldenburg.de/viridic/
VirClust v2	Moraru C.^51^	https://rhea.icbm.uni-oldenburg.de/virclust/

### Experimental model and study participant details

This research did not employ experimental models, and it did not involve animals, human subjects, plants, cell lines, or primary cell cultures.

### Method details

#### Biosafety approval

*B. pseudomallei* isolates were grown and tested under Biosafety Level 2 Enhanced (BSL-2+) containment procedures at the Department of Microbiology, Faculty of Medicine, Khon Kaen University. This research project was approved by the Institutional Biosafety Committees of the Faculty of Medicine Siriraj Hospital, Mahidol University and the Faculty of Medicine, Khon Kaen University. The approval certificate numbers are SI 2020-003 and IBCKKU no. 2/2563, respectively.

#### *Burkholderia* spp. culture condition

*B. pseudomallei*, *B. thailandensis* and BTCV were cultured in Luria-Bertani (LB) broth (Criterion™, Hardy Diagnostics, USA) at 37°C. Mid-log phase cultures were obtained by inoculating 1% of an overnight culture into fresh LB broth and incubating at 37°C for 4 h until reaching an OD600–0.18 (∼10^8^ CFU/mL).

#### Soil sampling and *B. pseudomallei* isolation

Soil samples were collected from rice paddy fields in Ubon Ratchathani (50 samples) and Khon Kaen (50 samples) provinces, located in the northeast of Thailand, for *B. pseudomallei* isolation according to a previously described method.^16^ Briefly, 5 g of soil was mixed with 30 mL of sterile distilled water and incubated at 40°C for 48 h. After sedimentation, the supernatant was transferred to Ashdown’s selective enrichment broth^52^ and incubated for another 48 h. Enriched samples were plated onto Ashdown’s agar. Suspected *B. pseudomallei* colonies were identified using biochemical assays and confirmed using a latex agglutination test.^35^

#### *B. thailandensis* and BTCV isolation

*B. thailandensis* (40 isolates) and BTCV (20 isolates) were isolated from soils collected from both northeastern and central Thailand (Table S1) as reported in the previous study by Hantrakul et al.^6^ These bacteria were kindly provided by Prof. Direk Limmathurotsakul, MORU, Thailand.

#### *Burkholderia* phages isolation from soil

To isolate culturable phages, 2 g of soil was suspended in 2x LB broth supplemented with CaCl_2_ and inoculated with *B. pseudomallei* UB4 as a bacterial host for enrichment. The UB4 isolate was selected as the enrichment bacterial host because it does not contain any detectable inducible prophages under mitomycin C (MMC; Sigma Aldrich, USA) treatment, reducing the likelihood of interference from host-derived phages during isolation. Cultures were incubated at 37°C for 24 h before centrifugation and were filtered through a 0.45 μm membrane to remove bacterial cells. Filtrates were screened for phage presence using a spot assay.^17^ Clear zones on the cultured medium indicated phage activity. Phages were then purified using the double agar overlay plaque assay.^17^

#### Prophage induction from *Burkholderia* spp.

*B. pseudomallei*, *B. thailandensis*, and BTCV were cultured to the exponential phase in LB broth, followed by the addition of 1 μg/mL mitomycin C (MMC; Sigma Aldrich, USA) and incubation at 37°C for 6 h. After centrifugation, the supernatant was filtered and screened for induced prophages by a spot assay against a panel of *B. thailandensis* isolates including E159, E174, E264, E348, D1, and DW503 (Table S1). This assay was performed to identify susceptible host strains for each phage. Positive MMC-induced prophages identified by the spot assay were subsequently confirmed by the double agar overlay plaque assay.^17^

#### Phage nomenclature

Phage names in this study were assigned following the convention recommended by Adriaenssens and Brister (2017),^53^ in which phages are designated with the prefix vB_ (“virus of bacteria”), followed by an abbreviation of the bacterial host and an isolate-specific identifier. MMC-induced prophages derived from *B. thailandensis* and BTCV were named using the format vB_<bacterial isolate>, such as vB_E188 and vB_E555, indicating phages induced from *B. thailandensis* isolate E188 and BTCV isolate E555, respectively. Prophages induced from soil-isolated *B. pseudomallei* strains were designated vB_SBP<isolateID>, for example vB_SBP39, referring to a prophage induced from *B. pseudomallei* isolate 39. Free phages recovered from soil using *B. pseudomallei* as the enrichment host were named using the prefix vB_F<phage isolate>, such as vB_FUB33 and vB_FKKS1, representing free phages isolated from soil samples collected in Ubon Ratchathani (UB33) and Khon Kaen (KKS1), respectively.

#### Transmission electron microscopy (TEM)

Phage morphology was examined by TEM after negative staining.^54^ Purified phage suspensions (10^8^ particles/mL) were fixed with 2.5% glutaraldehyde and applied to Formvar/carbon-coated copper grids (200 mesh) (Sigma Aldrich, USA). After adsorption for 5 min, the grids were stained with 1% uranyl acetate. Images were captured using a JEM-1400 transmission electron microscope (JEOL, Japan) operating at 80 kV.

#### Phage DNA extraction, sequencing and genome comparative analysis

Phage genomic DNA was prepared following the same workflow described in our previous study,^17^ utilizing the Illumina DNA Library Prep kit (Illumina, Inc., USA) for library construction and the Illumina MiSeq for sequencing. Raw sequencing reads were processed by trimming low-quality bases with Sickle v1.33^36^ and Trimmomatic v0.39.^37^ Quality-filtered reads were then assembled *de novo* using both SPAdes v3.6.0^38^ and Unicycler v0.4.8.^39^ Open reading frames (ORFs) were predicted and annotated using Prokka v1.12^40^ against a custom database tailored to *Caudoviricetes*. To assess genome termini and packaging mechanisms, all assemblies were analyzed with PhageTerm v1.0.12.^41^ As no fixed termini were detected, genomes were oriented following established phage genomics conventions by placing the terminase (Ter) gene at the start position when present. For clades whose closest reference genomes initiated elsewhere, orientation was refined using conserved synteny with related phages in public databases. This standardized orientation was applied prior to all comparative and collinearity analyses.

Functional annotations of predicted proteins were assigned through homology searches against BLASTP (http://www.ncbi.nlm.nih.gov/BLAST), HHPred v2.08^42^ and InterPro v106.0.^43^ Circular phage genome maps were visualized using Artemis v17.0.1.^44^ To infer evolutionary relationships, whole-genome sequences were aligned using MAFFT v7.505^45^ under the auto-alignment setting, producing nucleotide alignments based on translated amino acid sequences. Phylogenetic trees were constructed using the neighbor-joining method^55^ and visualized with Interactive Tree of Life (iTOL) v6.5.8.^46^ For comparative genome analysis, Clinker v0.0.25^47^ was employed to examine gene synteny and modular conservation. To classify phages at the family level and explore proteomic diversity, hierarchical clustering was conducted using the Viral Proteomic Tree method (VipTree) v1.1.3.^48^

To validate taxonomic assignments and genomic relationships, three additional ICTV-aligned genome-based tools were applied: VICTOR v1,^49^ VIRIDIC v1.1,^50^ and VirClust v2.^51^ Intergenomic similarities and clustering patterns were evaluated against reference *Burkholderia* phages in GenBank. Taxonomic boundaries followed ICTV genomic thresholds: species ≥95% intergenomic similarity (≈Genome-BLAST Distance Phylogeny method (GBDP) ≤ 0.05), genus/subclade 70–95% (≈ GBDP ≤0.25), and family/clade 40–70% (≈ GBDP ≤0.45), in accordance with the updated ICTV classification framework.^56^

### Quantification and statistical analysis

No inferential statistical analyses were performed in this study. Sample sizes (*n*) represent the number of independent soil samples, bacterial isolates (Table 2), or phage genomes analyzed (Tables 1 and S1). Reported proportions and percentages were calculated directly from observed counts within each dataset (e.g., number of phages per clade divided by the total number analyzed) without replication-based statistical testing. Plaque morphology characteristics, including plaque size, clarity, and the presence or absence of halo-like features, were evaluated qualitatively based on repeated plaque assays. Phylogenomic clustering were assessed using whole-genome sequence comparisons, as described in the method details. All bioinformatic analyses were performed using publicly available software, with software versions described in the method details and key resources table.

## Supplemental information

Document S1. Figures S1–S5

Table S1. Genomic and host information of 82 *Burkholderia* phages analyzed in this study, including accession numbers, sources, propagation hosts, genomic features, and nucleotide BLAST results

Table S2. Classification of genomic termini and DNA packaging mechanisms determined using PhageTerm for 82 tailed phages

Table S3. Taxonomic clustering of the analyzed phages was determined using the Genome BLAST Distance Phylogeny (GBDP) approach as implemented in VICTOR, applying formulas D0, D4, and D6 for intergenomic distance calculation, related to Figure S1

Table S4. Pairwise intergenomic similarity matrix of *Burkholderia* phages calculated with VIRIDIC, related to Figure S2

Table S5. Protein-based clustering of Burkholderia phages generated with VirClust, related to Figure S3

Table S6. Accession information of *Burkholderia* phage sequences retrieved from public NCBI databases

Table S7. Predicted functions of phage accessory genes based on BLASTP, InterPro, and HHpred analyses
