# Supplementary material for: Genomic diversity and clade clustering of Burkholderia pseudomallei and B. thailandensis prophages with soil-derived phages
Source: iScience. 2026 Jan 9;29(2):114658. doi: 10.1016/j.isci.2026.114658 (PMC12860723; doi:10.1016/j.isci.2026.114658)
Supplement: Document S1. Figures S1–S5 [file mmc1.pdf]

iScience, Volume *29*

Supplemental information

Genomic diversity and clade clustering of *Burkholderia pseudomallei* and *B. thailandensis* prophages with soil-derived phages

Patoo Withatanung, Veerachat Muangsombut, Sujintana Janesomboon, Vanaporn Wuthiekanun, Premjit Amornchai, Sorujsiri Chareonsudjai, Dave J. Baker, Martha R.J. Clokie, Edouard E. Galyov, Ozan Gundogdu, and Sunee Korbsrisate

Figure S1

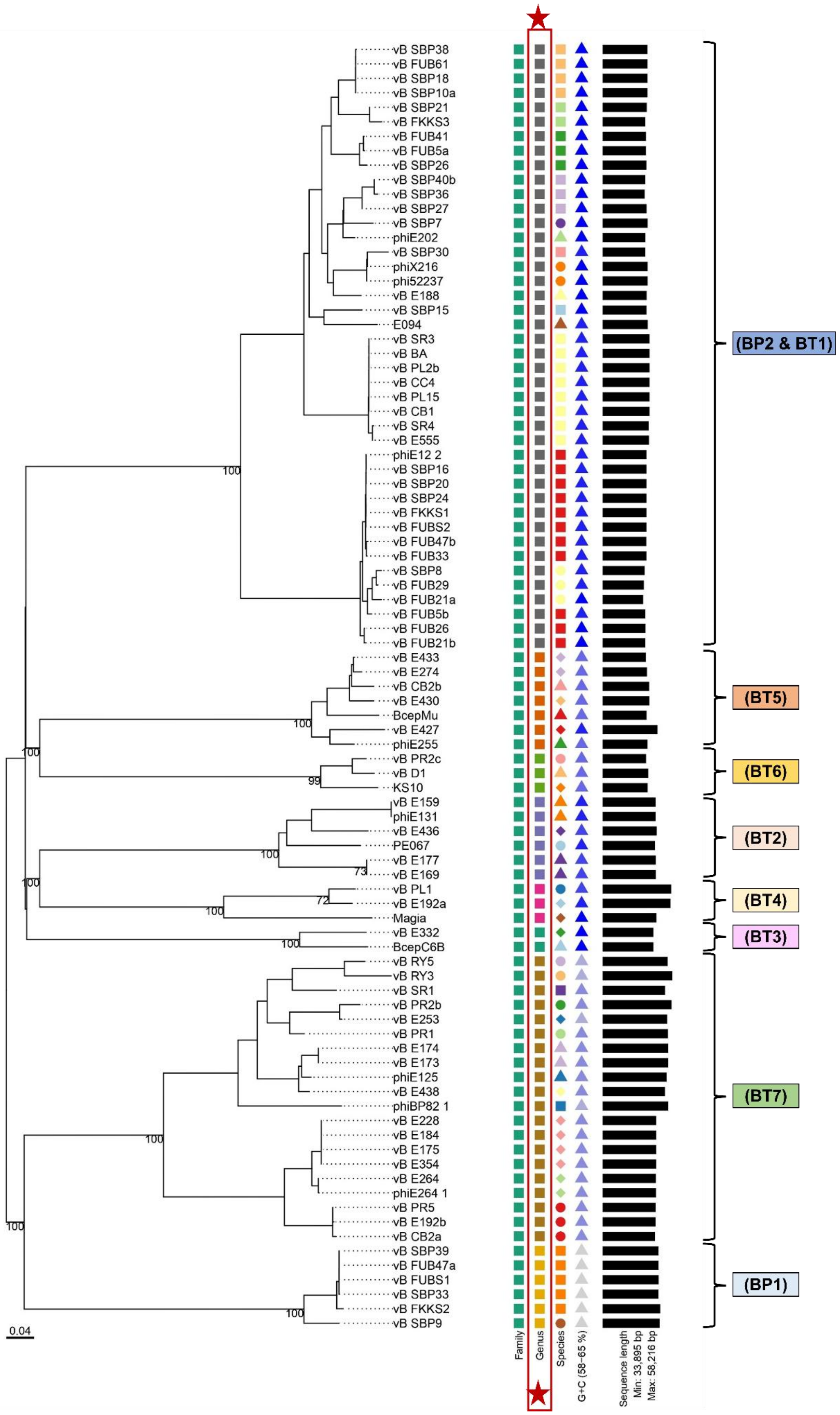
vB SBP38
vB FUB61
vB SBP18
vB SBP10a
vB SBP21
vB FKKS3
vB FUB41
vB FUB5a
vB SBP26
vB SBP40b
vB SBP36
vB SBP27
vB SBP7
phiE202
vB SBP30
phiX216
phi52237
vB E188
vB SBP15
E094
vB SR3
vB BA
vB PL2b
vB CC4
vB PL15
vB CB1
vB SR4
vB E555
phiE12 2
vB SBP16
vB SBP20
vB SBP24
vB FKKS1
vB FUBS2
vB FUB47b
vB FUB33
vB SBP8
vB FUB29
vB FUB21a
vB FUB5b
vB FUB26
vB FUB21b
vB E433
vB E274
vB CB2b
vB E430
BcepMu
vB E427
phiE255
vB PR2c
vB D1
KS10
vB E159
phiE131
vB E436
PE067
vB E177
vB E169
vB PL1
vB E192a
Magia
vB E332
BcepC6B
vB RY5
vB RY3
vB SR1
vB PR2b
vB E253
vB PR1
vB E174
vB E173
phiE125
vB E438
phiBP82 1
vB E228
vB E184
vB E175
vB E354
vB E264
phiE264 1
vB PR5
vB E192b
vB CB2a
vB SBP39
vB FUB47a
vB FUBS1
vB SBP33
vB FKKS2
vB SBP9
100
100
99
100
73
100
72
100
100
100
100
100
100
100
0.04
Family
Genus
Species
G+C (58–65 %)
Sequence length
Min: 33,895 bp
Max: 58,216 bp
(BP2 & BT1)
(BT5)
(BT6)
(BT2)
(BT4)
(BT3)
(BT7)
(BP1)

FIGURE S1 VICTOR GBDP phylogenomic tree of the *Burkholderia* phages and reference genomes. The tree was generated using the VICTOR Genome BLAST Distance Phylogeny (GBDP) method under the D0 formula, which represents overall nucleotide identity from BLAST high-scoring pairs and is recommended for species-genus-level resolution. Taxon IDs shown alongside the genomes are internal identifiers assigned by VICTOR to link each genome across the tree and distance matrices. Taxonomic boundaries follow ICTV-aligned thresholds: species ≥ 95% intergenomic similarity (≈ GBDP ≤ 0.05), genus 70-95% (≈ GBDP ≤ 0.25), and family 40-70% (≈ GBDP ≤ 0.45). Reference *Burkholderia* phages from GenBank were included as taxonomic anchors. The figure displays D0-based distances, while full D0, D4, and D6 distance matrices are provided in Table S3.

Figure S2

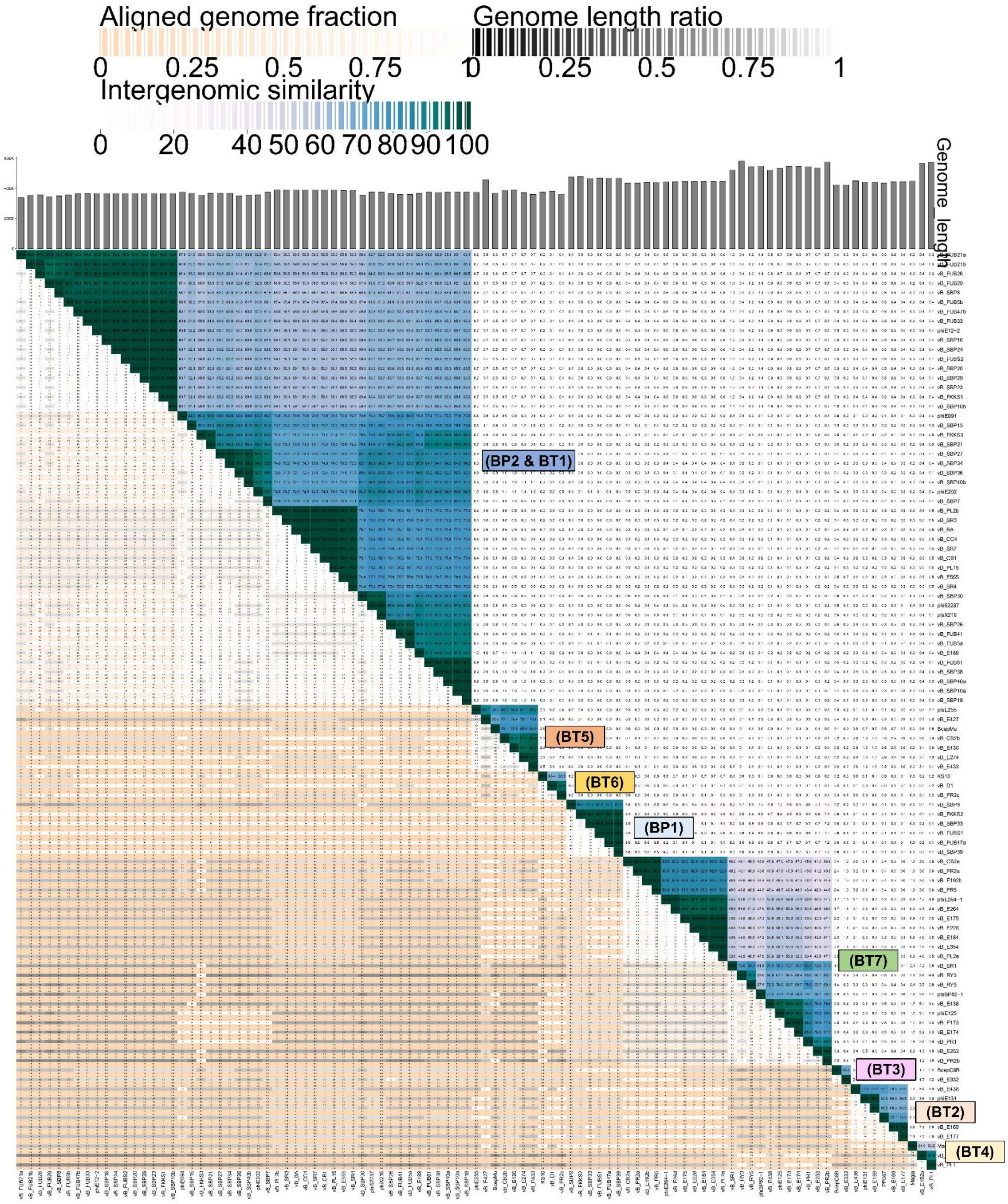

Aligned genome fraction
0
0.25
0.5
0.75
1
Genome length ratio
0
0.25
0.5
0.75
1
Intergenomic similarity
0
20
40 50 60 70 80 90 100
Genome_length
(BP2 & BT1)
(BT5)
(BT6)
(BP1)
(BT7)
(BT3)
(BT2)
(BT4)

FIGURE S2 VIRIDIC intergenomic similarity heatmap of *Burkholderia* phages together with reference genomes. The heatmap shows pairwise intergenomic similarity values of the phages on the right side using the VIRIDIC color scale, revealing eight well-defined clades. The aligned genome fraction and genome length ratio values are displayed on the left with their respective scales. Intergenomic similarity was calculated as 100 × (aligned length × average identity / smaller genome length) according to the VIRIDIC algorithm. Taxonomic thresholds applied for classification were: species ≥ 95%, genus (subclades) 70-95%, and family (clades) 40-70% intergenomic similarity. Clade assignments correspond to those recovered from the VICTOR GBDP phylogeny and were jointly used for species-, genus-, and family-level designations. Complete pairwise similarity scores are provided in Table S4.

Figure S3

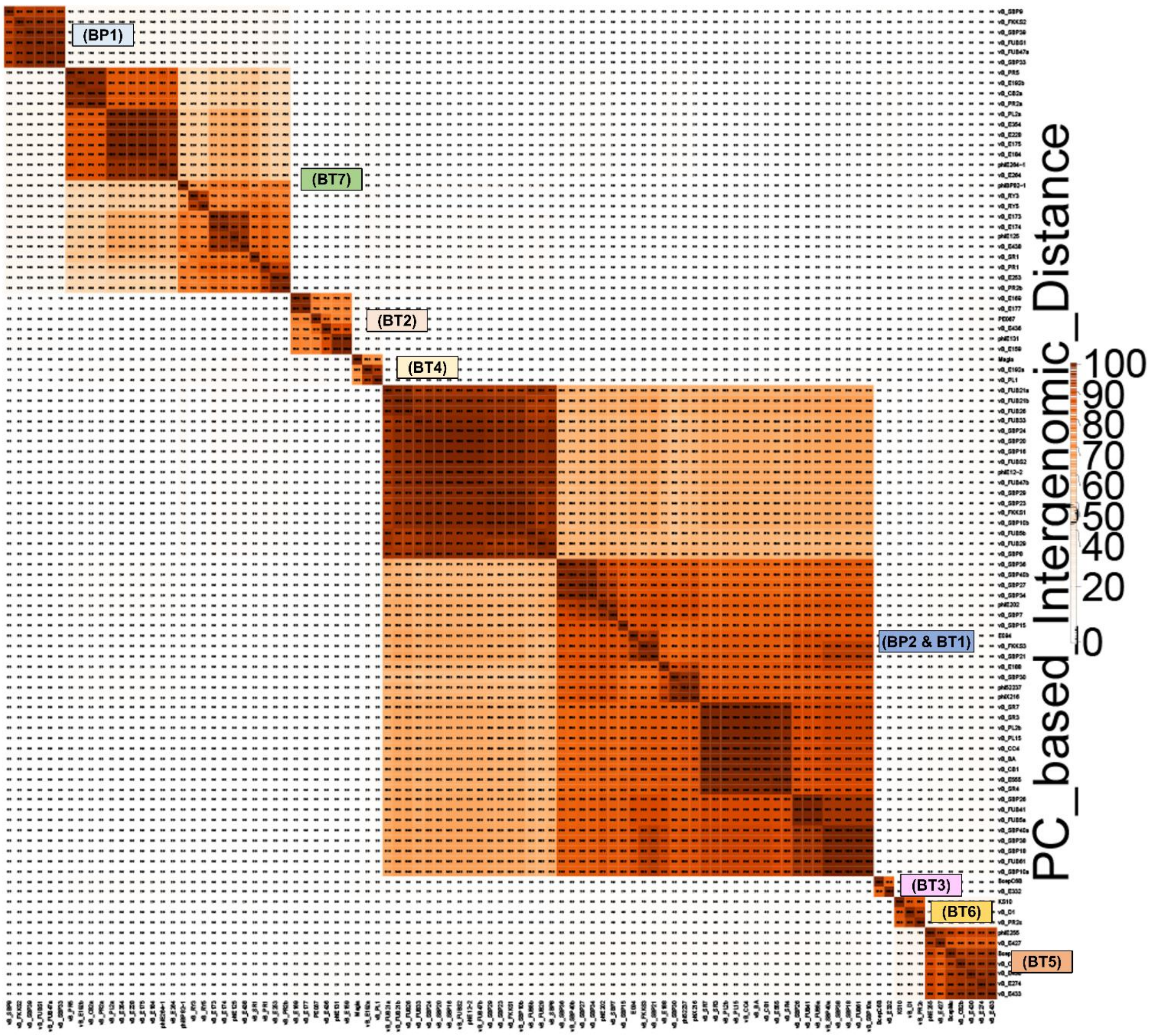

FIGURE S3 VirClust PC-based intergenomic distance heatmap of the *Burkholderia* phages and reference genomes. The heatmap shows pairwise PC-based intergenomic distances as calculated by VirClust. Darker colors indicate higher similarity, while lighter colors represent lower similarity. Pairwise shared protein content (%) was computed as the percentage of protein clusters present in both genomes relative to the smaller genome. Taxonomic thresholds applied for classification were: species ≥ 95%, genus (subclades) 70-95%, and family (clades) 40-70% based on intergenomic similarity used in conjunction with protein-content clustering. Complete PC-based distance values for all genome pairs are provided in Table S5.

Figure S4

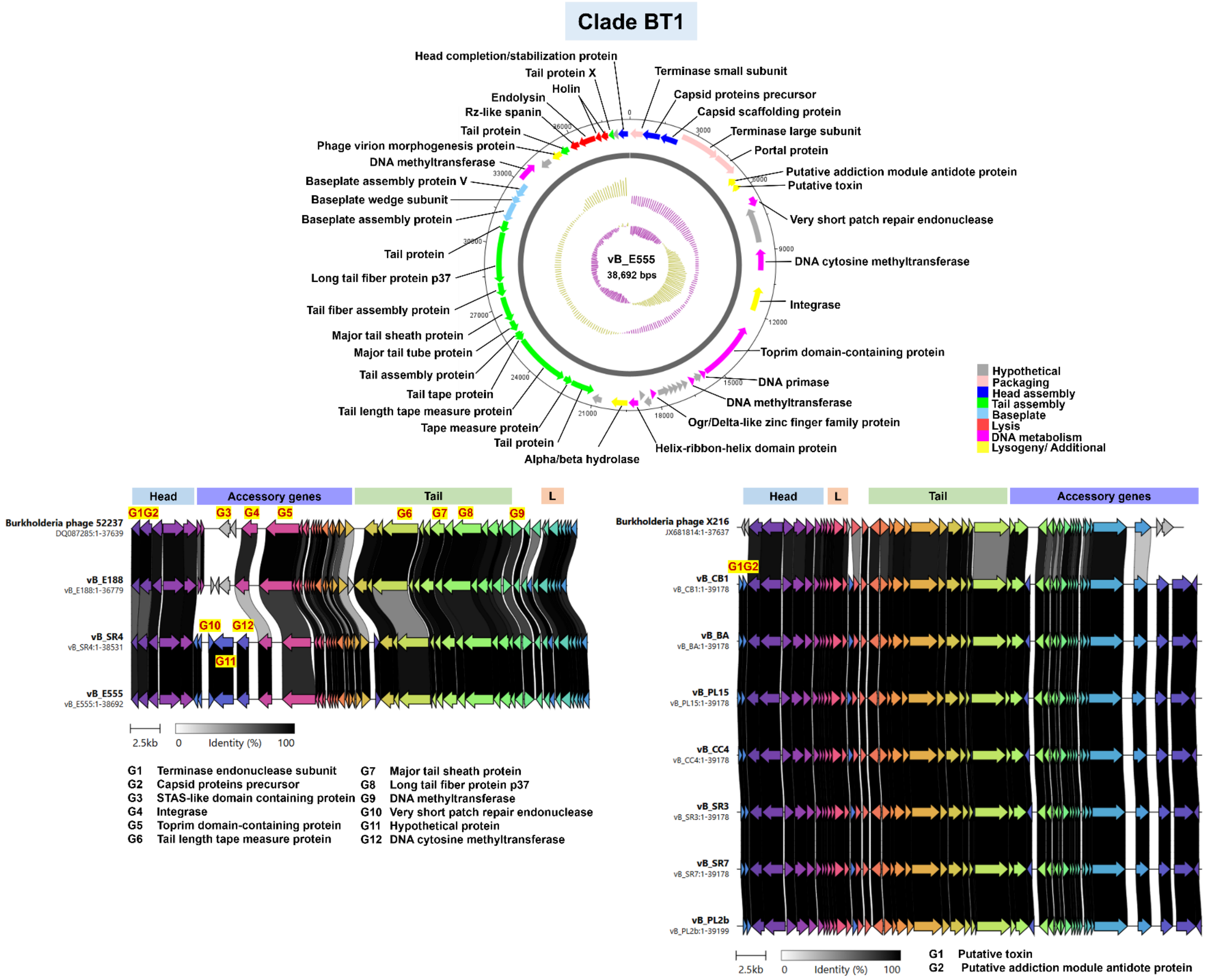

Clade BT1
Head completion/stabilization protein
Tail protein X
Holin
Endolysin
Rz-like spanin
Tail protein
Phage virion morphogenesis protein
DNA methyltransferase
Baseplate assembly protein V
Baseplate wedge subunit
Baseplate assembly protein
Tail protein
Long tail fiber protein p37
Tail fiber assembly protein
Major tail sheath protein
Major tail tube protein
Tail assembly protein
Tail tape protein
Tail length tape measure protein
Tape measure protein
Tail protein
Alpha/beta hydrolase
Terminase small subunit
Capsid proteins precursor
Capsid scaffolding protein
Terminase large subunit
Portal protein
Putative addiction module antidote protein
Putative toxin
Very short patch repair endonuclease
DNA cytosine methyltransferase
Integrase
Toprim domain-containing protein
DNA primase
DNA methyltransferase
Ogr/Delta-like zinc finger family protein
Helix-ribbon-helix domain protein
vB_E555
38,692 bps
Hypothetical
Packaging
Head assembly
Tail assembly
Baseplate
Lysis
DNA metabolism
Lysogeny/ Additional
Head
Accessory genes
Tail
L
Burkholderia phage 52237
DQ087285:1-37639
vB_E188
vB_E188:1-36779
vB_SR4
vB_SR4:1-38531
vB_E555
vB_E555:1-38692
G1G2
G3
G4
G5
G6
G7
G8
G9
G10
G12
G11
2.5kb
0
Identity (%)
100
G1 Terminase endonuclease subunit
G2 Capsid proteins precursor
G3 STAS-like domain containing protein
G4 Integrase
G5 Toprim domain-containing protein
G6 Tail length tape measure protein
G7 Major tail sheath protein
G8 Long tail fiber protein p37
G9 DNA methyltransferase
G10 Very short patch repair endonuclease
G11 Hypothetical protein
G12 DNA cytosine methyltransferase
Head
L
Tail
Accessory genes
Burkholderia phage X216
JX681814:1-37637
G1G2
vB_CB1
vB_CB1:1-39178
vB_BA
vB_BA:1-39178
vB_PL15
vB_PL15:1-39178
vB_CC4
vB_CC4:1-39178
vB_SR3
vB_SR3:1-39178
vB_SR7
vB_SR7:1-39178
vB_PL2b
vB_PL2b:1-39199
2.5kb
0
Identity (%)
100
G1 Putative toxin
G2 Putative addiction module antidote protein

FIGURE S4 Genomic and synteny analysis of *B. thailandensis* phages in clade BT1. Upper panels: Genome visualization shows the phage genome as a dark grey circle organized into eight functional modules (colored bars). The outer scale denotes genome position in bases, with 0 as the origin of replication. The inner circle displays GC skew (yellow: positive; purple: negative), and the outermost circle shows GC content (yellow: above average; purple: below average). **Lower panels:** Synteny analysis compares four genome modules: head, tail, lysis, and accessory, between phages in each clade and their corresponding reference phages from the GenBank database (phages 52237 and X216). ORFs are shown as arrows, colored by homologous gene clusters, with grey shading indicating amino acid identity (0-100%). Genes with notable variations among phages within each clade are annotated and highlighted in yellow.

Figure S5

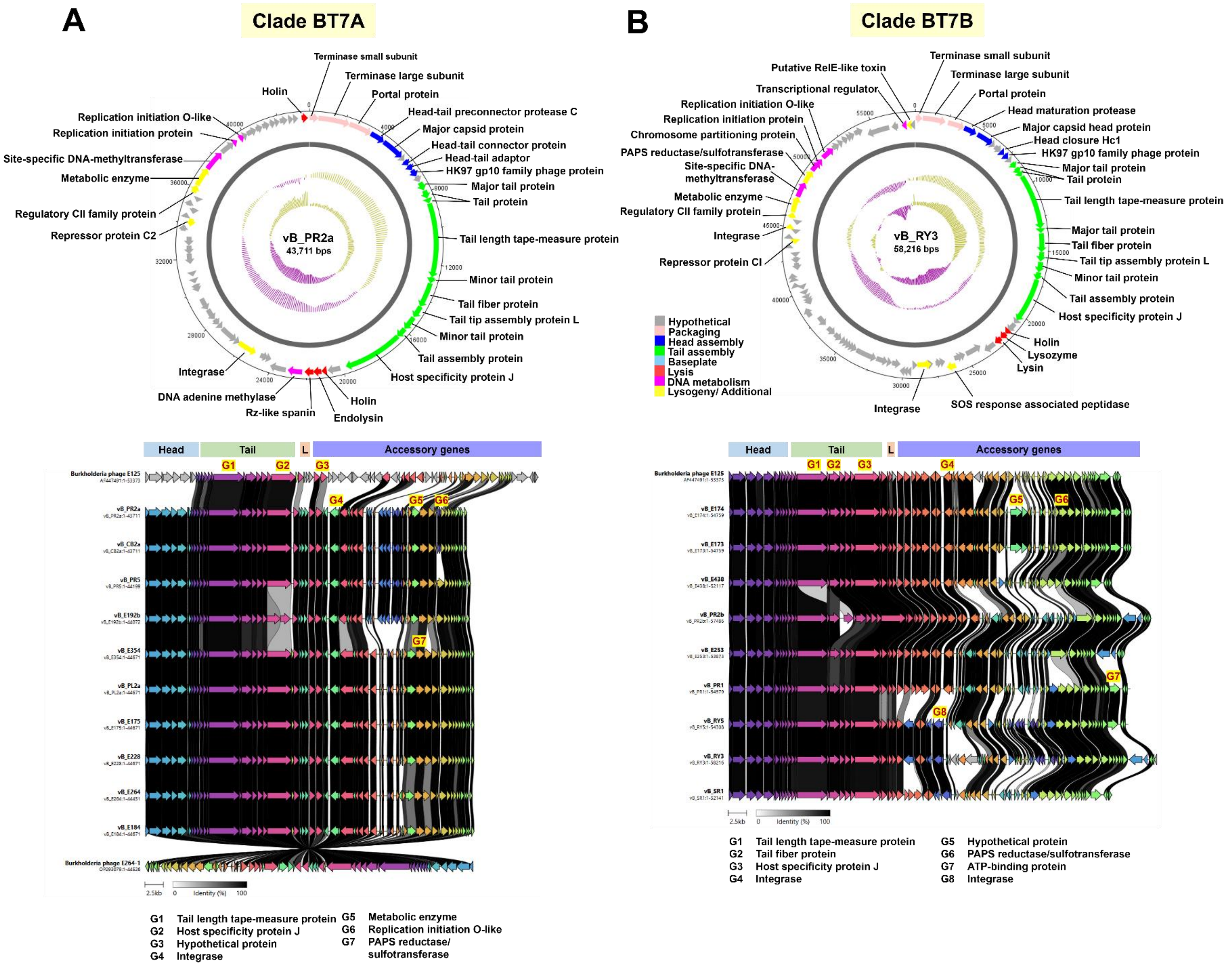
A
Clade BT7A
vB_PR2a
43,711 bps
Terminase small subunit
Terminase large subunit
Portal protein
Head-tail preconnector protease C
Major capsid protein
Head-tail connector protein
Head-tail adaptor
HK97 gp10 family phage protein
Major tail protein
Tail protein
Tail length tape-measure protein
Minor tail protein
Tail fiber protein
Tail tip assembly protein L
Minor tail protein
Tail assembly protein
Host specificity protein J
Holin
Endolysin
Rz-like spanin
DNA adenine methylase
Integrase
Repressor protein C2
Regulatory CII family protein
Metabolic enzyme
Site-specific DNA-methyltransferase
Replication initiation protein
Replication initiation O-like
Holin
Head
Tail
L
Accessory genes
Burkholderia phage E125
vB_PR2a
vB_CB2a
vB_PR5
vB_E192b
vB_E354
vB_PL2a
vB_E175
vB_E228
vB_E264
vB_E184
Burkholderia phage E264-1
2.5kb
Identity (%)
G1 Tail length tape-measure protein
G2 Host specificity protein J
G3 Hypothetical protein
G4 Integrase
G5 Metabolic enzyme
G6 Replication initiation O-like
G7 PAPS reductase/ sulfotransferase
B
Clade BT7B
vB_RY3
58,216 bps
Terminase small subunit
Terminase large subunit
Portal protein
Head maturation protease
Major capsid head protein
Head closure Hc1
HK97 gp10 family phage protein
Major tail protein
Tail protein
Tail length tape-measure protein
Major tail protein
Tail fiber protein
Tail tip assembly protein L
Minor tail protein
Tail assembly protein
Host specificity protein J
Holin
Lysozyme
Lysin
SOS response associated peptidase
Integrase
Repressor protein CI
Integrase
Regulatory CII family protein
Metabolic enzyme
Site-specific DNA-methyltransferase
PAPS reductase/sulfotransferase
Chromosome partitioning protein
Replication initiation protein
Replication initiation O-like
Transcriptional regulator
Putative RelE-like toxin
Hypothetical
Packaging
Head assembly
Tail assembly
Baseplate
Lysis
DNA metabolism
Lysogeny/ Additional
Head
Tail
L
Accessory genes
Burkholderia phage E125
vB_E174
vB_E173
vB_E438
vB_PR2b
vB_E253
vB_PR1
vB_RY5
vB_RY3
vB_SR1
2.5kb
Identity (%)
G1 Tail length tape-measure protein
G2 Tail fiber protein
G3 Host specificity protein J
G4 Integrase
G5 Hypothetical protein
G6 PAPS reductase/sulfotransferase
G7 ATP-binding protein
G8 Integrase

FIGURE S5 Genomic and synteny analysis of *B. thailandensis* phages in clade BT7. Representative phages from each clade are shown in (A) BT7A (vB_PR2a) and (B) BT7B (vB_RY3). **Upper panels:** The phage genome is shown as a dark grey circle organized into eight functional modules (colored bars). The outer scale denotes genome position in bases, with 0 as the origin of replication. The inner circle displays GC skew (yellow: positive; purple: negative), and the outermost circle shows GC content (yellow: above average; purple: below average). **Lower panels:** Synteny analysis compares four genome modules: head, tail, lysis, and accessory, between phages in this clade and their corresponding reference phages from the GenBank database (phage E125). ORFs are shown as arrows, colored by homologous gene clusters, with grey shading indicating amino acid identity (0-100%). Genes with notable variations among phages within the clade are annotated and highlighted in yellow.
